# HMGB1 aggravates neuronal ferroptosis and microglial inflammation in metabolic syndrome-associated cognitive impairment via the NRF2-autophagic flux axis

**DOI:** 10.1186/s43556-026-00548-8

**Published:** 2026-08-18

**Authors:** Xin Zhang, Yue Zhang, Meng-Meng Tang, Jia-Mei Zhang, Ya-Qin Xiao, Jian-Jun Yang, Cun-Ming Liu, Min Yu

**Affiliations:** 1 Department of Anesthesiology and Perioperative Medicine, The First Affiliated Hospital of Nanjing Medical University, Nanjing, 210029 China; 2 Department of Anesthesiology, The Fourth Affiliated Hospital of Nanjing Medical University, Nanjing, 210031 China; 3 Department of Anesthesiology, Jinling Hospital, Medical School of Nanjing University, Nanjing, 210002 China; 4 Department of Anesthesiology, Jiangsu Province Academy of Traditional Chinese Medicine, Nanjing, 210008 China; 5 Department of Anesthesiology, Pain and Perioperative Medicine, The First Affiliated Hospital of Zhengzhou University, Zhengzhou, 450000 China

**Keywords:** Metabolic syndrome, HMGB1, NRF2, Ferroptosis, Inflammation, Autophagic flux

## Abstract

Metabolic syndrome (MetS)-associated cognitive impairment is a growing global health crisis. The hippocampus is particularly vulnerable to metabolic stress, yet the precise molecular mechanisms linking systemic metabolic dysfunction to cognitive decline remain unclear. High-mobility group box 1 (HMGB1), a damage-associated molecule, is elevated in MetS. However, its specific role in simultaneously regulating hippocampal neuronal ferroptosis and microglial activation is poorly defined. Here we show that HMGB1 exacerbates MetS-associated cognitive impairment by promoting both neuronal ferroptosis and microglial inflammation. Using a high-fat high-glucose diet mouse model and primary co-cultures, we found that HMGB1 neutralization alleviated cognitive deficits, reduced neuronal ferroptosis, and suppressed microglial activation. Mechanistically, extracellular HMGB1 acts through neuronal toll-like receptor 4 (TLR4) to trigger the ubiquitination and degradation of nuclear factor erythroid 2-related factor 2 (NRF2). This HMGB1-induced loss of NRF2 impairs autophagic flux, thereby aggravating ferroptosis and neuroinflammation. Furthermore, in vitro conditioned medium experiments revealed a reciprocal pathological aggravation, where ferroptotic neurons and activated microglia both secrete HMGB1 to exacerbate each other's pathological state. These findings position HMGB1 as a pivotal mediator linking metabolic stress specifically to neuronal ferroptosis and neuroimmune activation. Targeting the HMGB1-NRF2-autophagic flux axis therefore offers a promising therapeutic strategy for mitigating cognitive impairment in patients with MetS.

**Supplementary Information:**

The online version contains supplementary material available at 10.1186/s43556-026-00548-8.

## Introduction

Metabolic syndrome (MetS), a cluster of metabolic abnormalities including central obesity, hyperglycemia, dyslipidemia, and hypertension, affects nearly one-third of the global adult population [1, 2]. Accumulating clinical evidence indicates that MetS is associated with a high risk of cognitive impairment [3, 4] which significantly compromises patients' quality of life. The hippocampus, a pivotal brain region for learning and memory, is particularly vulnerable to metabolic stress [5, 6]. However, the specific mechanisms linking metabolic syndrome to cognitive decline remain incompletely elucidated, hindering the development of targeted therapeutic strategies.

Recent studies indicate that ferroptosis, an iron-dependent form of lipid peroxidation, is deeply implicated in hippocampal neuronal injury under metabolic dysfunction [7, 8]. Concurrently, microglial activation and the secretion of pro-inflammatory cytokines are consistently observed in the hippocampus of MetS subjects [9, 10]. High-mobility group box 1 (HMGB1), a highly conserved nuclear protein, is released upon cellular stress to exert extracellular effects primarily via toll-like receptor 4 (TLR4) and receptor for advanced glycation end products (RAGE) [11, 12]. Elevated HMGB1 levels are observed in individuals with MetS [13, 14], and it is known to drive ferroptosis and inflammation in various injury models [15–17]. Nevertheless, whether HMGB1 concurrently modulates hippocampal neuronal ferroptosis and microglial inflammation in MetS remains undefined.

Nuclear factor erythroid 2-related factor 2 (NRF2) is a master transcription factor regulating cellular defense against oxidative stress, inflammation, and ferroptosis. In the brain, NRF2 activation protects neurons in models of neurodegenerative diseases and ischemic stroke [18–20]. Intriguingly, HMGB1 inhibition leads to increased NRF2 expression and activation in diabetic nephropathy and sepsis-induced lung injury, implying a potential HMGB1-NRF2 regulatory link [21, 22]. However, whether this regulatory link exists in the hippocampus under MetS conditions is unknown. Furthermore, HMGB1 and NRF2 are both implicated in regulating autophagy [23–27], a process often impaired in MetS that contributes to tissue inflammation [28, 29]. It remains to be clarified whether HMGB1 regulates NRF2 to disrupt autophagic flux in the context of MetS-associated cognitive impairment.

In this study, we aimed to investigate the role of HMGB1 in MetS-associated cognitive impairment and its underlying molecular mechanisms. Here we show that HMGB1 exacerbates hippocampal cognitive deficits by simultaneously promoting neuronal ferroptosis and microglial inflammation. Using both high-fat high-glucose diet-fed mice and simulated in vitro co-culture models, we demonstrated that extracellular HMGB1 promotes NRF2 ubiquitination and degradation primarily via TLR4, leading to impaired autophagic flux. Furthermore, in vitro conditioned medium experiments suggested a reciprocal pathological aggravation between ferroptotic neurons and activated microglia mediated by HMGB1. Overall, our findings validate HMGB1 as a key therapeutic target and map the HMGB1-NRF2-autophagic flux axis as a critical mechanism driving cognitive decline in MetS.

## Results

### Inhibition of HMGB1 alleviates MetS-induced NRF2 reduction, ferroptosis, inflammation and cognitive impairment in mice

To verify if targeting HMGB1 mitigates MetS-associated cognitive impairment and explore the possible mechanisms, we established a MetS mouse model using a high-fat high-glucose diet (HFHGD), which successfully induced systemic metabolic dysfunction (Fig. S1). Mice were then administered an HMGB1 neutralizing antibody (HMGB1 Ab). We subsequently evaluated hippocampal HMGB1 and NRF2 protein levels, neuronal ferroptosis, microglial inflammation, and cognitive behaviors. Neuronal ferroptosis was assessed by neuronal survival via Nissl staining, alongside Fe2⁺, malondialdehyde (MDA), and acyl-CoA synthetase long-chain family member 4 (ACSL4) levels. Microglial inflammation was indicated by ionized calcium-binding adapter molecule 1 (IBA-1), TNF-α, and IL-6 expression. Cognitive behaviors were evaluated via novel object recognition (NORT) and Morris water maze (MWM) tests.

**Fig. 1:**
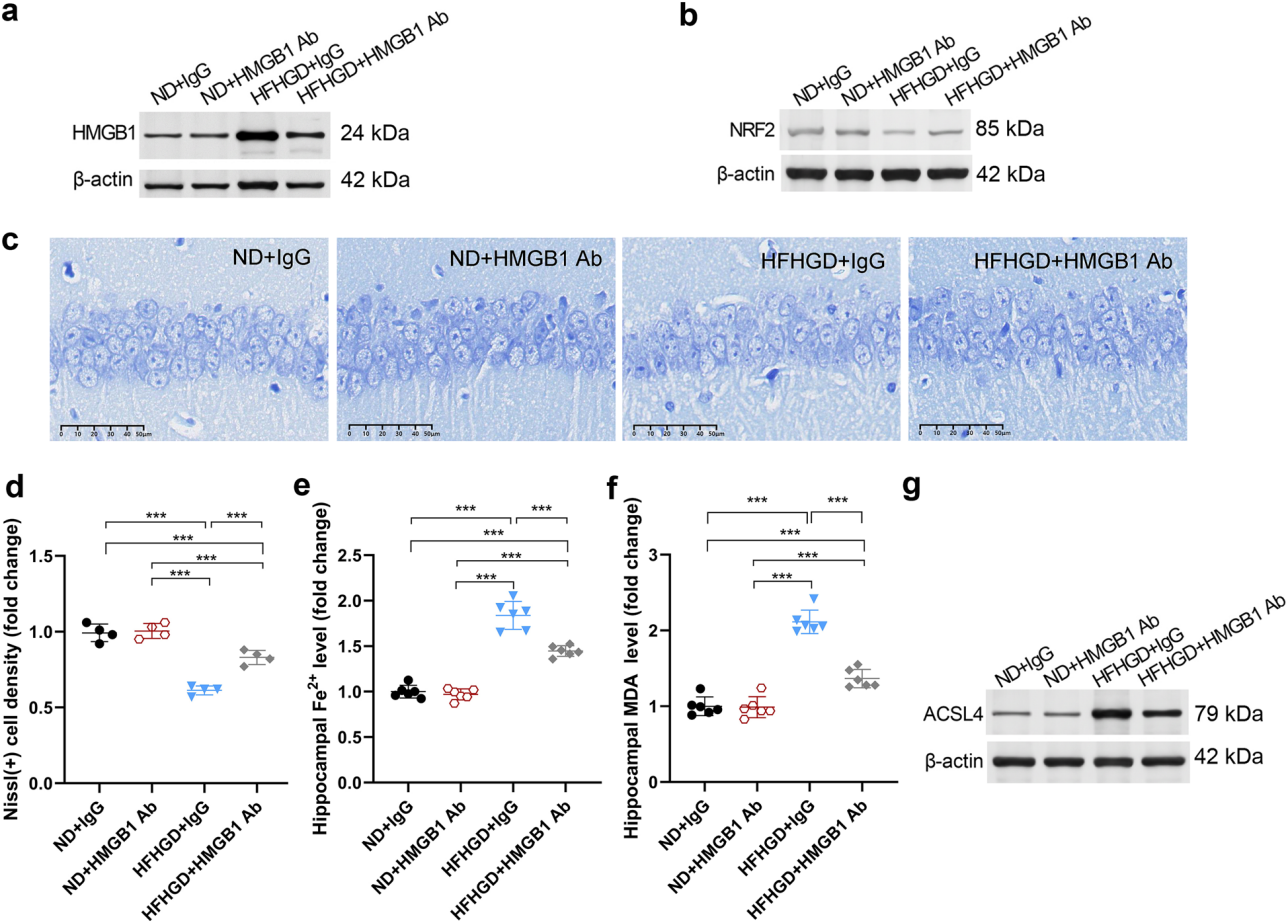
HMGB1 blockade alleviates MetS-induced hippocampal NRF2 reduction and neuronal ferroptosis. **a**, **b** Representative Western blot images of HMGB1 (**a**) and NRF2 (**b**) protein expression in the hippocampus (*n* = 4 mice per group). **c**, **d** Representative Nissl staining images (scale bar = 50 μm) (**c**) and quantification of Nissl-positive cell density (**d**) (*n* = 4 mice per group). **e**, **f** Intracellular Fe^2^⁺ (**e**) and MDA (**f**) levels in the hippocampus (*n* = 6 mice per group). **g** Representative Western blot image of ACSL4 protein expression (*n* = 4 mice per group). (Quantification of the immunoblots is provided in Fig. S2). Data are presented as mean ± SD. Statistical significance was evaluated using one-way ANOVA followed by Bonferroni post-hoc tests. * *P* < 0.05, ** *P* < 0.01, *** *P* < 0.001

**Fig. 2:**
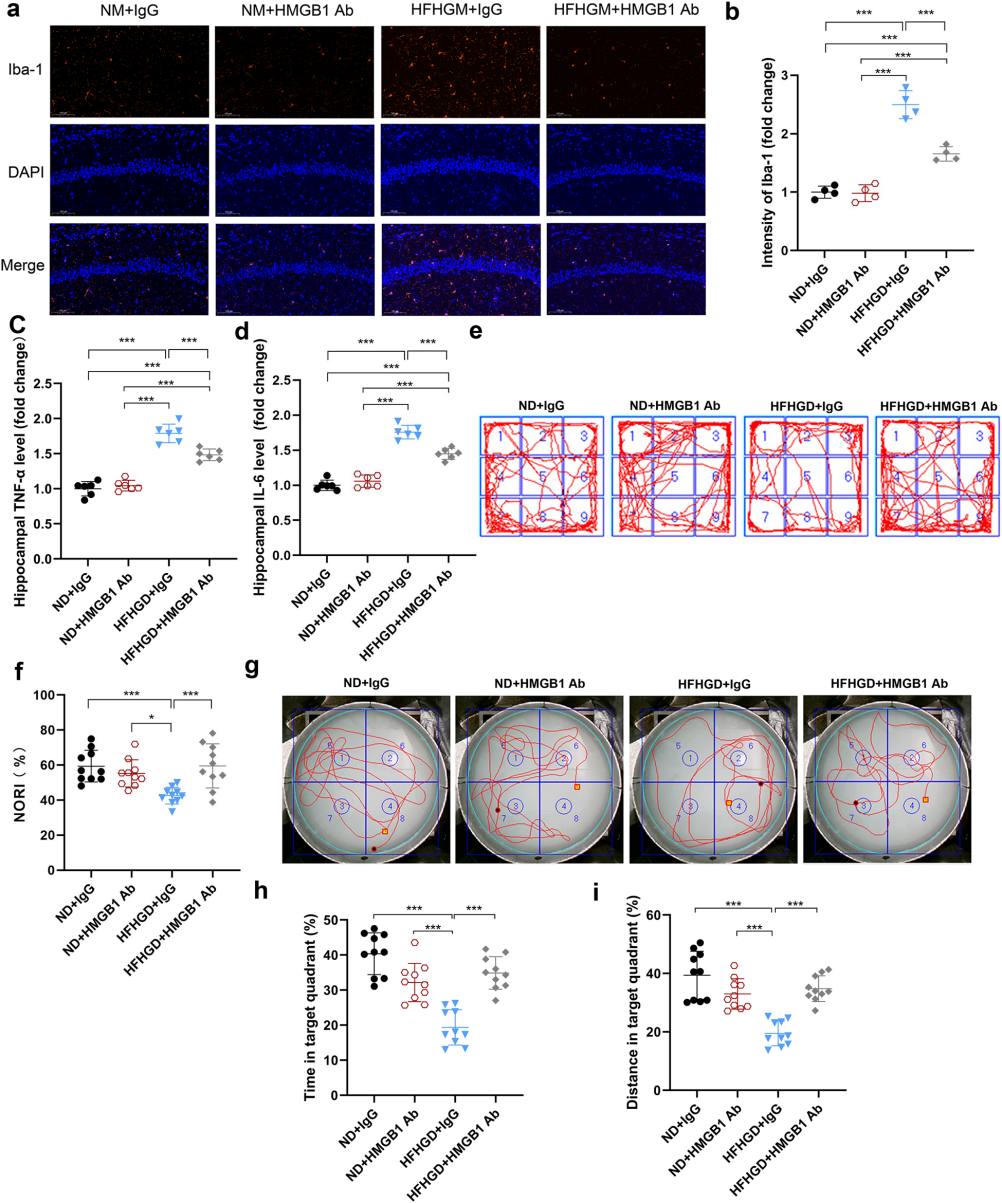
HMGB1 blockade suppresses MetS-induced microglial inflammation and improves cognitive impairment. **a**, **b** Representative immunofluorescent staining (bar = 100 μm) (**a**) and intensity of IBA-1 (**b**) (*n* = 4 mice per group). **c**, **d** TNF-α (**c**) and IL-6 (**d**) protein levels in the hippocampus measured by ELISA (*n* = 6 mice per group). **e**, **f** Tracing graphs (**e**) and NORI (**f**) of the mice in the NORT (*n* = 10 mice per group). **g, h, i** Tracing graphs (**g**), percentages of time spent (**h**), and swimming distance in the target quadrant (**i**) during the MWM test (*n* = 10 mice per group). Data are presented as mean ± SD. Statistical significance was evaluated using one-way ANOVA followed by Bonferroni post-hoc tests. * *P* < 0.05, ** *P* < 0.01, *** *P* < 0.001

For the normal diet (ND) baseline controls, detailed phenotypic results showed that ND + IgG and ND + HMGB1 Ab groups exhibited comparable hippocampal HMGB1 and NRF2 levels (quantifications in Fig. S2), intact neuronal density with low baseline ferroptotic indicators, as well as normal inflammatory and cognitive metrics. Conversely, HFHGD feeding induced a significant increase in HMGB1 and a decrease in NRF2 (Fig. 1a, b), alongside severe neuronal ferroptosis (evidenced by marked neuronal disorganization in Nissl staining and elevated Fe^2^⁺, MDA, and ACSL4) (Fig. 1c-g). Importantly, HMGB1 Ab intervention in HFHGD-fed mice robustly reversed these ferroptotic and molecular alterations, although not fully returned them to ND levels. Similarly, the HFHGD-induced microglial activation (Fig. 2a-d) and spatial/recognition cognitive impairments (Fig. 2e-i) were significantly mitigated by HMGB1 Ab treatment. Collectively, these in vivo findings suggest that HMGB1 neutralization effectively alleviates HFHGD-induced ferroptosis and neuroinflammation, translating to improved cognitive function.

### HMGB1 aggravates neuronal ferroptosis and microglial activation in simulated MetS microenvironment

**Fig. 3:**
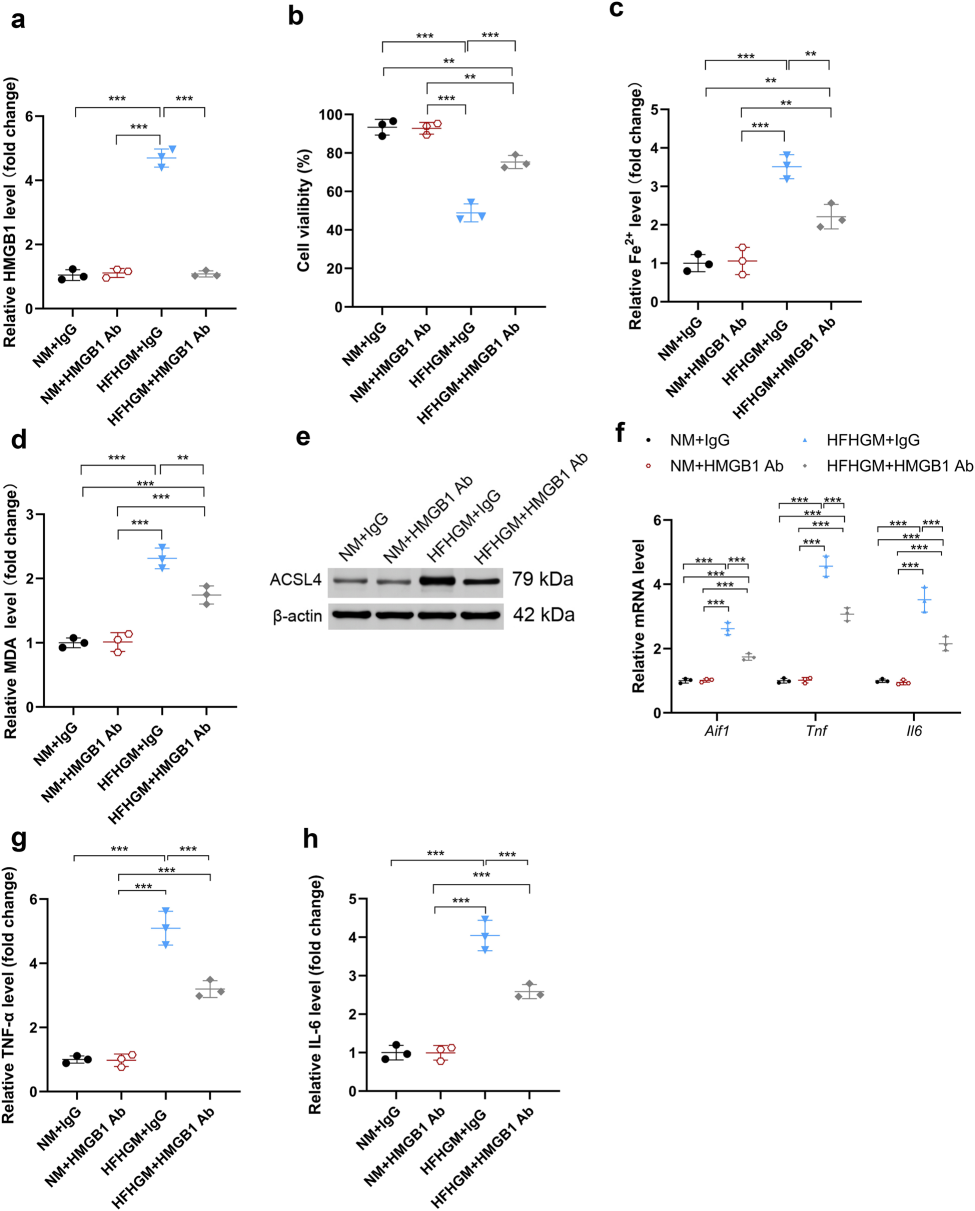
HMGB1 is a key regulator driving neuronal ferroptosis and microglial activation under HFHGM stress. **a** HMGB1 protein levels in the co-culture medium of mouse primary neurons and microglia measured by ELISA. **b** Viability of neurons from the murine co-culture system following normal medium (NM) or HFHGM treatment with or without HMGB1 neutralizing antibody. **c**, **d** Intracellular Fe^2^⁺ (**c**) and MDA (**d**) levels in primary neurons from the co-culture system. **e** Representative Western blot image of ACSL4 protein expression in primary neurons from the co-culture system. **f** Relative mRNA levels of *Aif1* (IBA-1), *Tnf* and *Il6* in primary microglia from the co-culture system. **g**, **h** Secreted TNF-α (**g**) and IL-6 (**h**) protein levels in the co-culture medium. (Quantification of the immunoblots is provided in Fig. S2). Data are presented as mean ± SD of three independent experiments (*n* = 3). Statistical significance was evaluated using one-way ANOVA followed by Bonferroni post-hoc tests. * *P* < 0.05, ** *P* < 0.01, *** *P* < 0.001

To verify the regulatory role of HMGB1 in a MetS-mimicking microenvironment, we cultured primary mouse neurons and microglia in normal medium (NM) or high-fat high-glucose medium (HFHGM, optimized in Fig. S3), supplemented with control IgG or HMGB1 Ab. We found that HMGB1 secretion was significantly elevated in the HFHGM-treated group compared to NM controls, an effect successfully suppressed by HMGB1 Ab (Fig. 3a). While NM controls exhibited normal baseline metrics, HFHGM exposure triggered pronounced neuronal ferroptosis (decreased cell viability; increased Fe^2^⁺, MDA, and ACSL4) and microglial inflammation (elevated IBA-1, TNF-α, and IL-6). These ferroptotic and inflammatory effects were markedly attenuated by the addition of HMGB1 Ab, though differences persisted compared to NM groups (Fig. 3b-h).

Consistently, *Hmgb1* knockdown (KD) via CRISPR/Cas9 (efficiency validated in Fig. S4) yielded similar protective outcomes against neuronal ferroptosis and microglial inflammation in this co-culture system (Fig. S5). These results indicate that extracellular HMGB1 acts as a key driver exacerbating neuronal ferroptosis and microglial inflammation under simulated MetS conditions.

### HMGB1 mediates the reciprocal aggravation between neuronal ferroptosis and microglial inflammation

**Fig. 4:**
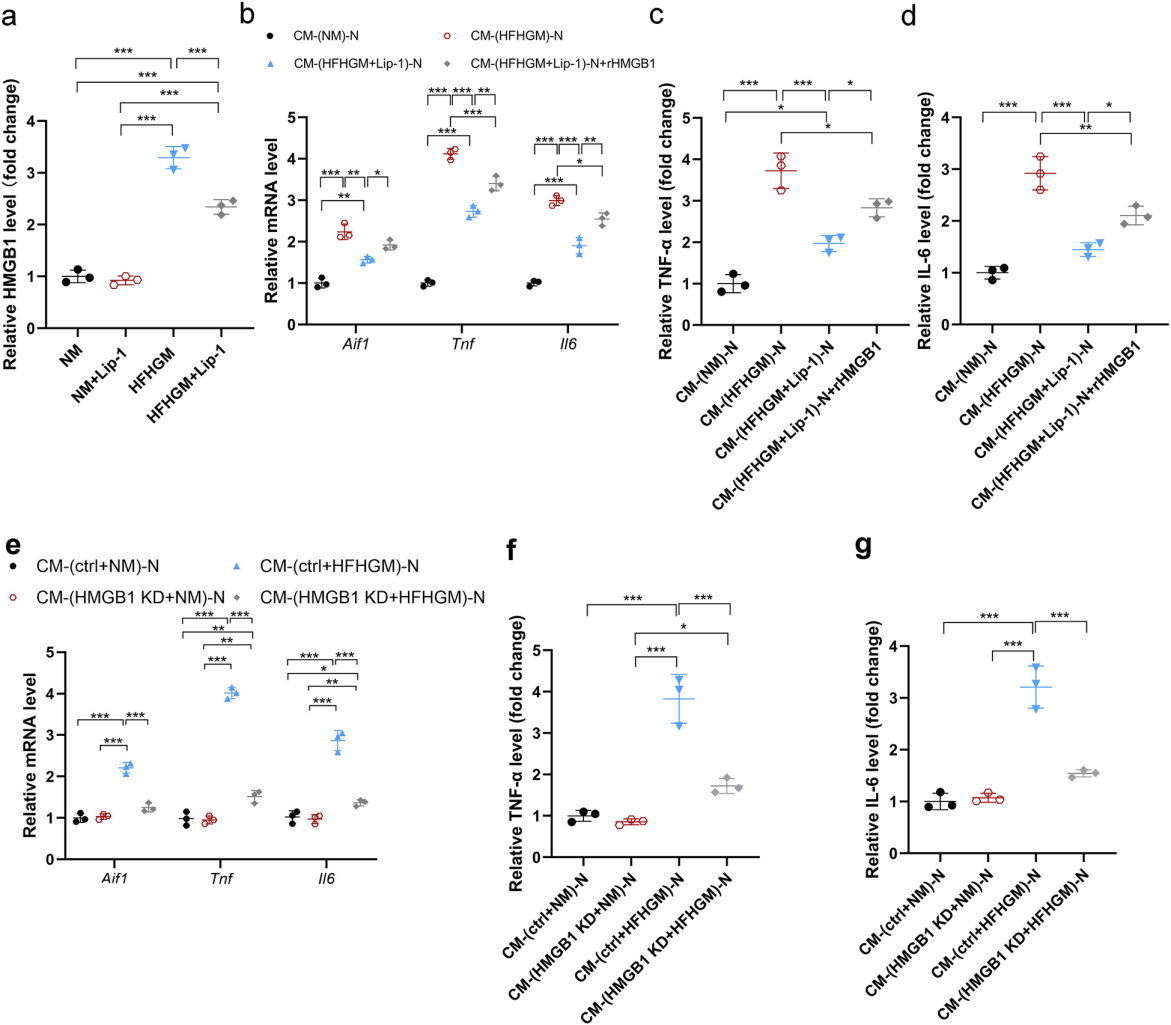
HMGB1 mediates microglial inflammation triggered by neuronal ferroptosis. **a** HMGB1 protein levels in the culture medium of primary neurons treated with normal medium (NM) or HFHGM in the presence or absence of the ferroptosis inhibitor Lip-1. **b**, **c**, **d** Relative *Aif1* (IBA-1), *Tnf* and *Il6* mRNA levels (**b**) and secreted TNF-α (**c**) and IL-6 (**d**) protein levels in primary microglia. Microglia were incubated with neuron-derived conditioned medium (CM-N) from the corresponding experimental groups in (a), with the final group supplemented with exogenous recombinant HMGB1 (rHMGB1). **e**, **f**, **g** Relative *Aif1* (IBA-1), *Tnf* and *Il6* mRNA levels (**e**) and secreted TNF-α (**f**) and IL-6 (**g**) protein levels in primary microglia treated with CM from *Hmgb1*-KD or control neurons. Data are presented as mean ± SD of three independent experiments (*n* = 3). Statistical significance was evaluated using one-way ANOVA followed by Bonferroni post-hoc tests. * *P* < 0.05, ** *P* < 0.01, *** *P* < 0.001

**Fig. 5:**
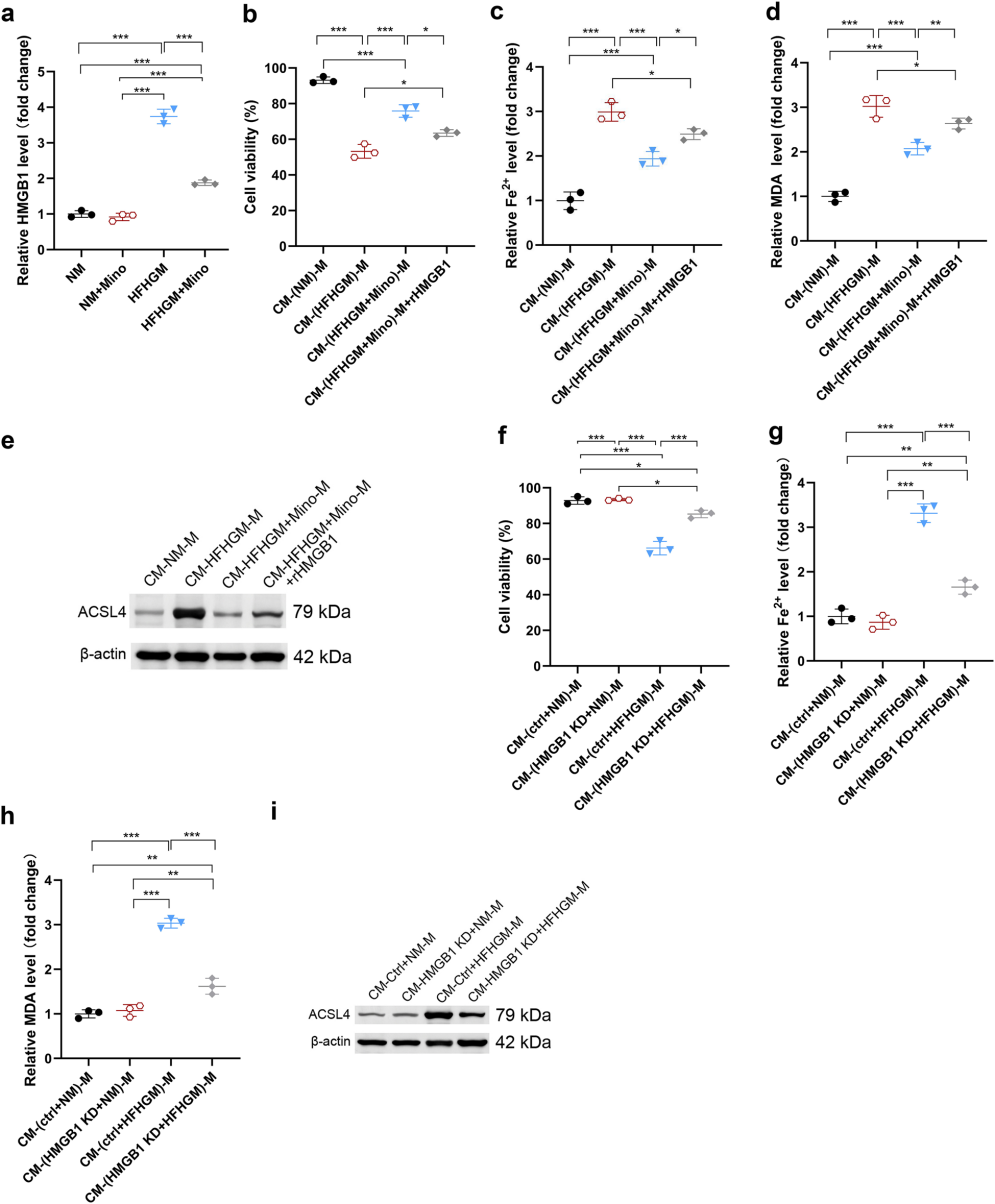
Microglia-derived HMGB1 reciprocally exacerbates neuronal ferroptosis.** a** HMGB1 protein level in the culture medium of primary microglia treated with NM or HFHGM in the presence or absence of the microglial activation inhibitor Minocycline (Mino). **b**, **c**, **d**, **e** Cell viability (**b**), intracellular Fe^2^⁺ (**c**) and MDA (**d**) levels, and representative Western blot image of ACSL4 protein expression (**e**) in primary neurons. Neurons were incubated with microglia-derived conditioned medium (CM-M) collected from the corresponding experimental groups in (**a**), with the final group supplemented with exogenous rHMGB1. **f**, **g**, **h**, **i** Cell viability (**f**), intracellular Fe^2^⁺ (**g**), MDA (**h**) levels, and representative Western blot image of ACSL4 protein expression (**i**) in primary neurons treated with CM derived from *Hmgb1*-KD or control microglia. (Quantification of the immunoblots is provided in Fig. S2). Data are presented as mean ± SD of three independent experiments (*n* = 3). Statistical significance was evaluated using one-way ANOVA followed by Bonferroni post-hoc tests. * *P* < 0.05, ** *P* < 0.01, *** *P* < 0.001

To explore whether HMGB1 mediates the pathological interaction between neuronal ferroptosis and microglial inflammation, we utilized conditioned medium (CM) crossover experiments incorporating specific inhibitors (Liproxstatin-1 [Lip-1] or Minocycline [Mino], optimized in Fig. S6), *Hmgb1* KD, and recombinant HMGB1 (rHMGB1). In the neuron-to-microglia paradigm, HFHGM-exposed neurons released significantly more HMGB1, which was partially abrogated by the ferroptosis inhibitor Lip-1 (Fig. 4a). CM from HFHGM-exposed neurons (CM-N) triggered severe microglial inflammation. Crucially, CM from Lip-1-treated neurons elicited a significantly milder inflammatory response; however, supplementing this medium with rHMGB1 reactivated the microglia (Fig. 4b-d). Furthermore, CM derived from *Hmgb1*-KD or HMGB1 Ab-neutralized neurons failed to provoke robust microglial inflammation (Fig. 4e-g; Fig. S7a-c). In the reciprocal microglia-to-neuron paradigm, HFHGM-activated microglia secreted high levels of HMGB1, suppressed by Mino (Fig. 5a). Neurons incubated with this microglia-derived CM (CM-M) underwent profound ferroptosis. Blocking microglial activation with Mino generated a CM that induced significantly less neuronal ferroptosis, but exogenous rHMGB1 reinstated the toxicity (Fig. 5b-e). Similarly, reducing microglial HMGB1 release protected recipient neurons from CM-induced ferroptosis (Fig. 5f-i; Fig. S7d-f). Together, HMGB1 mediates a reciprocal pathological aggravation: ferroptotic neurons release HMGB1 to provoke microglia, while activated microglia secrete HMGB1 to worsen neuronal ferroptosis.

### HMGB1 promotes NRF2 ubiquitination and degradation, and impairs nuclear translocation, involving TLR4 signaling

**Fig. 6:**
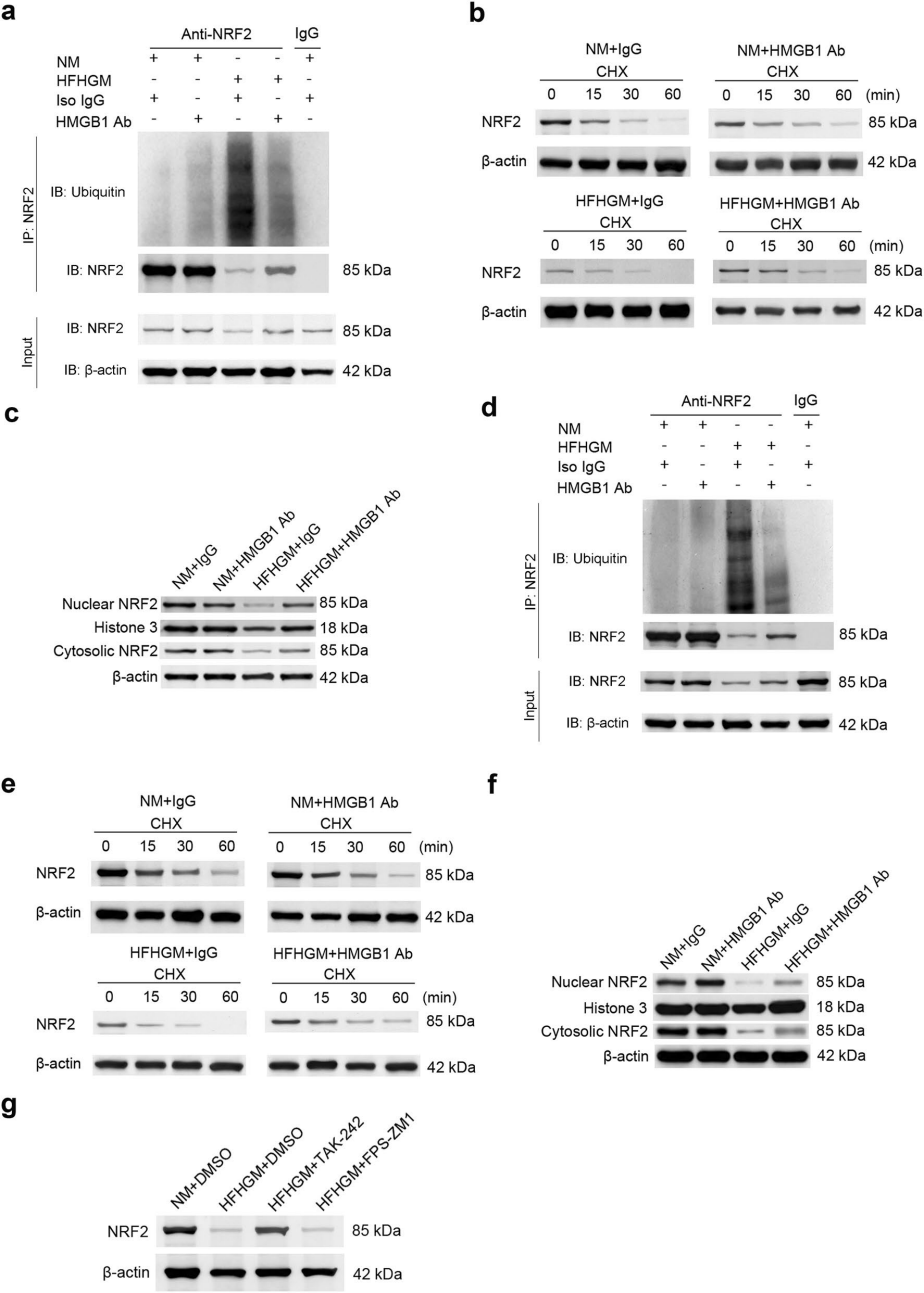
HMGB1 promotes NRF2 ubiquitination and degradation, and impairs nuclear translocation, involving TLR4 signaling. **a** Co-immunoprecipitation (Co-IP) analysis of NRF2 ubiquitination in primary neurons treated with or without HMGB1 Ab. **b** Cycloheximide (CHX) chase assay assessing NRF2 protein stability in primary neurons. **c** Representative Western blot images of nuclear and cytosolic fractions evaluating NRF2 nuclear translocation in primary neurons. **d** Co-IP analysis of NRF2 ubiquitination in primary microglia. **e** CHX chase assay assessing NRF2 protein stability in primary microglia. **f** Evaluation of NRF2 nuclear translocation in primary microglia. **g** Representative Western blot images and quantification of NRF2 protein levels in primary neurons pre-treated with the TLR4 inhibitor TAK-242 or the RAGE inhibitor FPS-ZM1 for 1 h prior to HFHGM exposure. (Quantification of the Western blots and Co-IP assays is provided in Fig. S2). All experiments were independently repeated 3 times

To elucidate the downstream molecular basis and upstream receptor by which extracellular HMGB1 drives pathology, we assessed NRF2 stability via co-immunoprecipitation (Co-IP), cycloheximide (CHX) chase assays, and nuclear/cytosolic fractionation, along with pharmacological receptor blockades (TAK-242 and FPS-ZM1). Co-IP and CHX analyses revealed that HFHGM exposure significantly promoted NRF2 ubiquitination and accelerated its degradation in both primary neurons and microglia compared to NM controls, subsequently reducing nuclear NRF2 translocation. Importantly, HMGB1 neutralization effectively suppressed NRF2 ubiquitination, stabilized the protein, and partially rescued its nuclear accumulation in both cell types (Fig. 6a-f, with quantitative bar graphs presented in Fig. S2). To identify the upstream receptor, we observed that pharmacological blockade of TLR4 (TAK-242), but not RAGE (FPS-ZM1), significantly prevented the HFHGM-induced reduction of NRF2 protein levels in neurons (Fig. 6g). These data demonstrate that extracellular HMGB1 compromises NRF2 stability and nuclear translocation, a process shown to involve TLR4 signaling in neurons.

### HMGB1 impairs NRF2-mediated autophagic flux to regulate neuronal ferroptosis and microglial inflammation

**Fig. 7:**
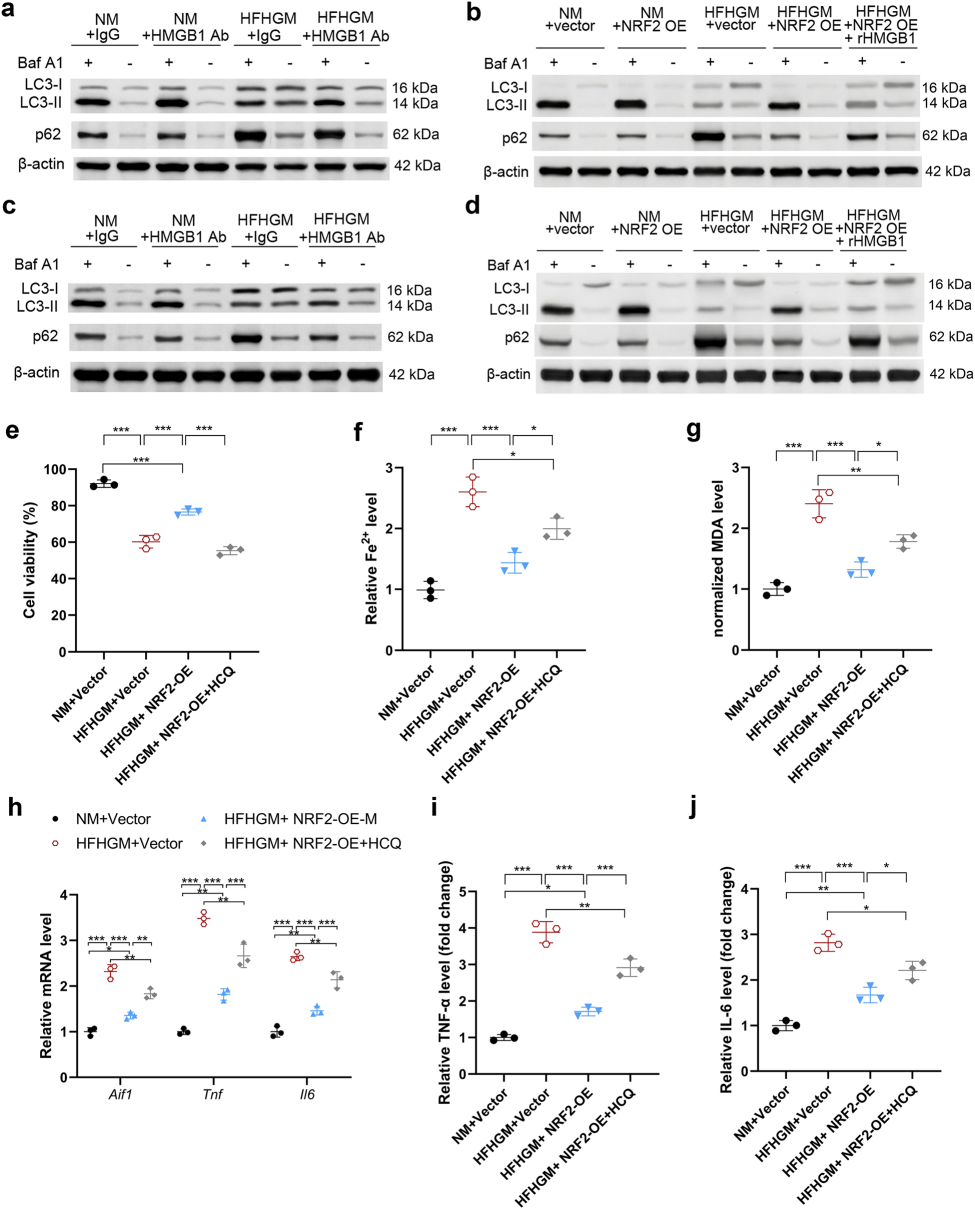
HMGB1 impairs NRF2-mediated autophagic flux, which is essential for regulating neuronal ferroptosis and microglial inflammation. **a** Representative Western blot images of LC3-II and p62 protein expression in primary neurons treated with or without HMGB1 Ab, in the presence or absence of Bafilomycin A1 (BafA1). **b** Representative Western blot images of LC3-II and p62 protein expression in neurons subjected to lentiviral *Nrf2* overexpression (OE) with or without rHMGB1 treatment. **c** Representative Western blot images of LC3-II and p62 protein expression in primary microglia treated with or without HMGB1 Ab and BafA1. **d** Representative Western blot images of LC3-II and p62 protein expression in microglia subjected to *Nrf2* OE with or without rHMGB1 treatment. (Quantification of the immunoblots is provided in Fig. S2). **e** Viability of primary neurons subjected to *Nrf2* OE and treated with or without the autophagy inhibitor hydroxychloroquine (HCQ). **f**, **g** Intracellular Fe^2^⁺ (**f**) and MDA (**g**) levels in neurons following *Nrf2* OE and HCQ treatment. **h** Relative *Aif1* (IBA-1), *Tnf* and *Il6* mRNA levels in primary microglia following *Nrf2* OE and HCQ treatment. **i**, **j** TNF-α (**i**) and IL-6 (**j**) protein levels in the culture medium of microglia following *Nrf2* OE and HCQ treatment. The experiments were repeated 3 times independently. Data are presented as mean ± SD (*n* = 3). Statistical significance was evaluated using one-way ANOVA followed by Bonferroni post-hoc tests. * *P* < 0.05, ** *P* < 0.01, *** *P* < 0.001

To determine whether HMGB1-induced NRF2 dysfunction impairs autophagic flux, and whether this mechanism dictates the downstream pathologies, we assessed autophagic markers (LC3-II/p62) with bafilomycin A1 (Baf A1), and performed functional rescue assays using lentiviral *Nrf2* overexpression (*Nrf2* OE, efficiency validated in Fig. S4) combined with the autophagy inhibitor hydroxychloroquine (HCQ). Under HFHGM conditions, primary cells exhibited an abnormal basal accumulation of both LC3-II and p62, indicating a severe blockade at the autophagic degradation stage. HMGB1 neutralization successfully improved autophagic flux, as evidenced by a robust further accumulation of LC3-II upon Baf A1 treatment (Fig. 7a, c). Similarly, *Nrf2* OE effectively reversed the HFHGM-induced autophagic impairment (alleviating the abnormal basal accumulation and improving Baf A1-induced flux), a protective effect that was significantly antagonized by rHMGB1 treatment (Fig. 7b, d; quantifications in Fig. S2). Regarding functional outcomes, *Nrf2* OE significantly attenuated HFHGM-induced neuronal ferroptosis (improving viability and decreasing Fe^2^⁺ and MDA) and suppressed microglial inflammatory responses (reducing *Aif1*, *Tnf*, and *Il6* mRNA and secreted cytokines). Notably, HCQ co-treatment substantially abolished these neuroprotective and anti-inflammatory benefits provided by *Nrf2* OE (Fig. 7e-j). Collectively, these findings confirm that extracellular HMGB1 exacerbates neuronal ferroptosis and microglial inflammation specifically by disrupting the NRF2-autophagic flux axis.

## Discussion

This study identifies HMGB1 as a key driver of MetS-associated cognitive impairment, with its effects mediated through the HMGB1-NRF2 axis. By validating HMGB1 as a critical regulator and NRF2 as a downstream hub, our work advances mechanistic understanding and provides a translatable therapeutic target for this growing clinical challenge.

In our study, we focused on HMGB1 due to its strong clinical relevance in metabolic disorders. Although public datasets specifically profiling HMGB1 in MetS-associated cognitive impairment are currently lacking, HMGB1 is recognized as a highly sensitive and specific circulating biomarker for MetS, outperforming traditional markers like IL-6 and adiponectin [13, 30]. Furthermore, HMGB1 levels are markedly elevated in patients with obesity and type 2 diabetes, and its association with cognitive deficits in diabetic populations strongly supports its relevance to MetS-related brain pathology [31–33]. Our in vivo findings successfully bridge this clinical correlation to mechanistic causality. To establish a highly susceptible baseline model for investigating this axis, we strategically utilized female mice. This design choice was driven by clinical epidemiological data indicating a higher MetS prevalence in women [1, 34–36], coupled with solid experimental evidence showing that the female hippocampus exhibits a heightened molecular sensitivity to high-fat diet-induced metabolic insults. This sex-specific vulnerability manifests as more severe cognitive impairment and pronounced microglial activation [37, 38], largely driven by distinct metabolic network reprogramming [39]. At the biological level, the systemic metabolic derangements in MetS serve as persistent stressors, which can drive the release of danger-associated molecular patterns (DAMPs). By leveraging this highly responsive female model, we confirmed that hippocampal HMGB1 is significantly upregulated in HFHGD-fed mice, accompanying definitive hallmarks of both neuronal ferroptosis and microglial activation. Notably, targeted neutralization of HMGB1 effectively alleviated these cognitive and pathological impairments without inducing noticeable side effects in normal-diet controls. This disease-specificity suggests a favorable therapeutic window for HMGB1 blockade compared to therapies broadly suppressing basal immune homeostasis [40].

Crucially, our work dissects the HMGB1-mediated pathological interaction between neuronal ferroptosis and microglial inflammation, an area often neglected by prior studies that viewed these as isolated events. Using conditioned medium paradigms, we established a reciprocal pathological aggravation. Ferroptotic neurons actively release HMGB1 to stimulate resting microglia; in parallel, activated microglia secrete HMGB1 that further sensitizes neurons to ferroptotic cell death. This finding updates the traditional neuroinflammatory model, in which microglia passively react to dead neurons, by demonstrating that HMGB1 serves as an active mediator capable of exacerbating both pathologies in a reciprocal manner [41, 42]. Functionally, this reciprocal interaction suggests that metabolic insults may progressively worsen neuroinflammation and cellular injury, potentially contributing to the chronic nature of cognitive decline in MetS patients.

Beyond delineating this cell–cell interaction, we clarified the downstream molecular mechanisms. We provide direct evidence that extracellular HMGB1 accelerates the ubiquitination and rapid degradation of intracellular NRF2, while concurrently impairing its nuclear translocation. Furthermore, our pharmacological blockade assays demonstrate that HMGB1-induced NRF2 protein depletion is mediated, at least in part, by TLR4 signaling in neurons. This HMGB1-driven loss of NRF2 functionally impairs the autophagic machinery, leading to a profound reduction in autophagic clearance (indicated by the pathological co-accumulation of LC3-II and p62).

We therefore confirmed that restoring NRF2 expression (*Nrf2* OE) successfully unblocked this autophagic flux, which subsequently dismantled both neuronal ferroptosis and microglial inflammation. To definitively establish causality, we utilized the specific autophagy inhibitor HCQ. Co-treatment with HCQ substantially abolished the neuroprotective and anti-inflammatory benefits provided by *Nrf2* OE. This compelling evidence confirms that autophagy is not merely an epiphenomenon, but rather the indispensable downstream effector of NRF2's neuroprotection. These findings are consistent with previous reports highlighting the indispensable role of the NRF2-autophagy axis in combating neurodegenerative stressors [43–47]. By positioning HMGB1 upstream of this axis, our data outline a pathway through which extracellular danger signals can compromise intracellular defense mechanisms.

Clinically, our findings address an urgent unmet need: MetS affects over 1 billion individuals worldwide, and its association with cognitive decline represents a growing public health crisis [48, 49]. HMGB1 is a highly druggable target: although HMGB1 neutralizing antibodies has not been reported in patients, the specific small-molecule inhibitors such as glycyrrhizin have demonstrated safety in clinical trials [50]. Therefore, intercepting this HMGB1-mediated interaction may offer a targeted strategy to simultaneously mitigate neuronal ferroptosis and progressive neuroinflammation associated with Mets-associated cognitive impairment.

Despite these promising findings, our study has limitations. First, while our conditioned medium experiments suggest a reciprocal interplay, definitive proof of a causal cycle in vivo would require sophisticated cell-type-specific inducible knockout models. Second, although we demonstrated that blocking TLR4 prevents NRF2 degradation in neurons, we cannot entirely rule out potential auxiliary roles of other receptors. Third, although our use of female mice provided a highly susceptible model to delineate the HMGB1-NRF2-autophagy mechanism, we acknowledge that significant sex differences exist in metabolism and neuroinflammation. Consequently, our current findings cannot be directly generalized to male subjects. Future studies incorporating both male and ovariectomized models will be essential to fully validate the sex universality of this pathological axis.

**Fig. 8:**
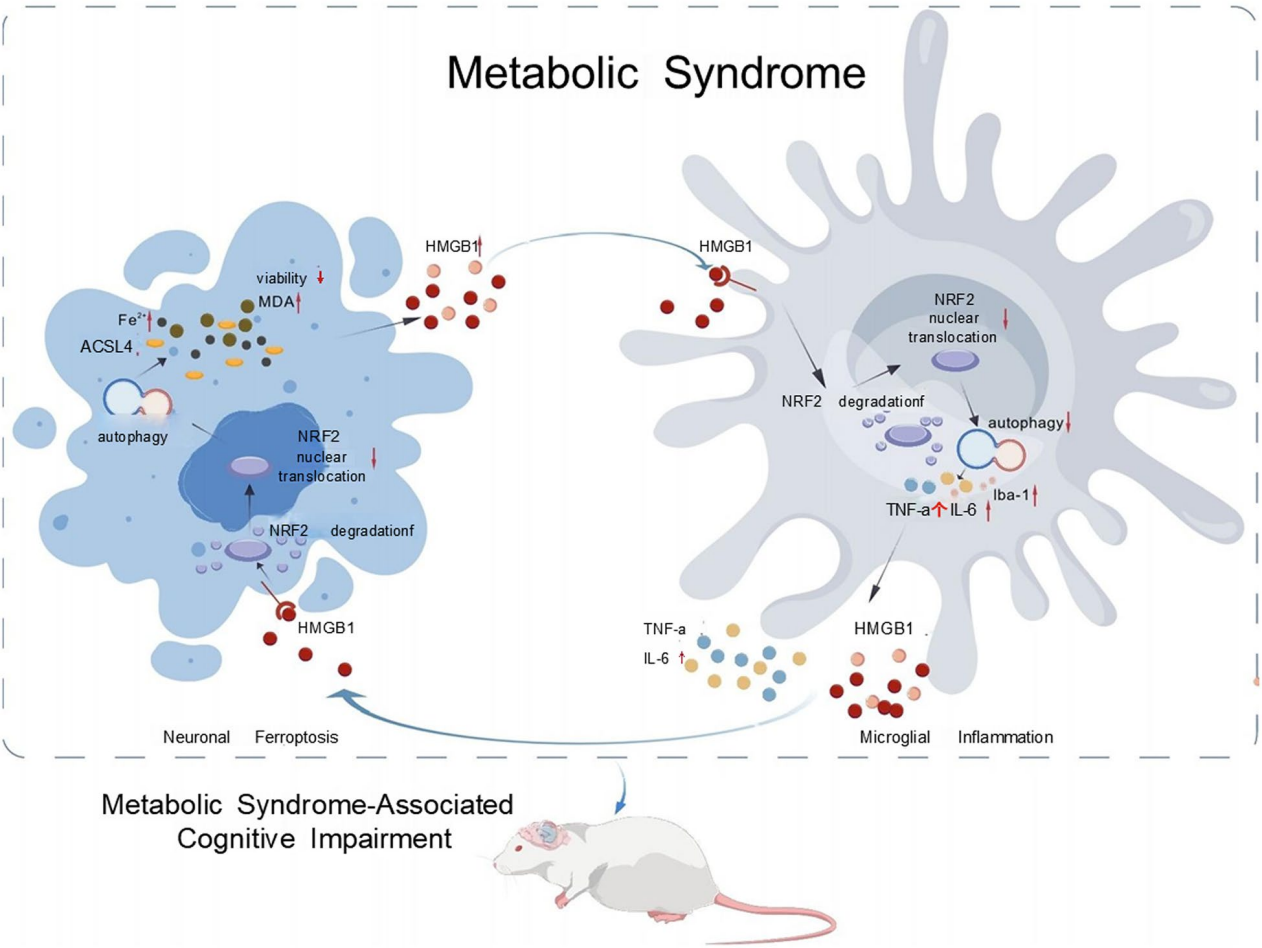
Schematic diagram of the proposed mechanism. In MetS microenvironment, hippocampal neurons undergo ferroptosis and release HMGB1, while activated microglia also secrete HMGB1. Extracellular HMGB1 acts via cell-surface receptors (primarily TLR4) to promote NRF2 degradation, which consequently reduces its nuclear translocation. The resultant loss of functional NRF2 impairs autophagic flux, leading to a reciprocal aggravation that further exacerbates neuronal ferroptosis (manifested as decreased viability, increased levels of Fe^2^⁺, MDA and ACSL4) and microglial inflammation (manifested as increased IBA-1, TNF-α and IL-6 levels). Inhibiting HMGB1 interrupts this pathological interplay by improving NRF2 stability and autophagic flux, ultimately alleviating cognitive impairment. The schematic diagram was created using BioGDP (https://biogdp.com)

In conclusion, we position HMGB1 as a central regulator of MetS-related cognitive impairment. Our study demonstrates that HMGB1 exacerbates the reciprocal aggravation between neuronal ferroptosis and microglial inflammation by modulating NRF2 stability and autophagic flux (Fig. 8). Validating HMGB1 as a disease-specific therapeutic target offers a new approach for the millions of MetS patients at risk of cognitive decline, with future work focused on refining mechanistic details and advancing clinical translation.

## Materials and methods

### Animals and treatments

Six-week-old female C57BL/6 J mice (Vital River Laboratories) were housed in the Animal Facility of Nanjing Medical University. Female mice were selected for this model to leverage their pronounced susceptibility to metabolic stress-induced neuroinflammation (detailed biological rationale is provided in the Discussion section). The mice were randomly divided to receive different dietary regimens, which were maintained for 16 weeks: a normal diet (ND) consisting of standard rodent chow (16% kcal from fat) and water, or a high-fat high-glucose diet (HFHGD) comprising high-fat chow (45% kcal from fat) with free access to 10% glucose solution. After 8 weeks of feeding, the mice were intraperitoneally injected with 5 mg/kg HMGB1 neutralizing antibody (HMGB1 Ab) (Duoneng-Bio, China) or IgG control once every three days. Ultimately, the animals were divided into 4 groups (*n* = 10 per group): ND + IgG, ND + HMGB1 Ab, HFHGD + IgG, HFHGD + HMGB1 Ab.

At the end of the experiment, the metabolic measurements were performed (supplementary materials). Following anesthesia, the mice’s brains were harvested and the hippocampi were isolated for subsequent analyses.

### Novel objective recognition test (NORT)

Each mouse was placed in an open-field apparatus with two identical objects (in Zone 1 and Zone 3) for 10 min for familiarization. After a 2 h interval, one of the objects was replaced with a new object (in Zone 7) differing in color, shape and position, and the mouse was allowed to explore for 5 min. The New Object Recognition Index (NORI) was calculated as (exploration time of new object)/(exploration time of old object + exploration time of new object) × 100%.

### Morris water maze (MWM) test

A round tank filled with opaque liquid was divided into four quadrants. The mice underwent four 60-s training sessions daily for 4 days, searching for the underwater escape platform in the target quadrant (Quadrant 1). Mice failing to find the platform within 60 s were guided to it for a 30-s rest. On the 5th day, the platform was removed and the mice swam freely for 60 s. The percentage of the time spent and the swimming distance in the target quadrant were measured.

### Nissl staining

Nissl staining was used to detect the surviving neurons. The brains of the mice were fixed, embedded and cut into 20 μm coronal cryostat sections, which were stained with Nissl staining solution (Beyotime, China) for 5 min at 37 °C.

### Immunofluorescent staining

The immunofluorescent staining for ionized calcium binding adaptor molecule-1 (IBA-1) was applied to detect the activated microglia in mice hippocampus. The cryostat sections were incubated with primary antibody for IBA-1 (Beyotime, China) at 4 °C overnight, followed by CY3-conjugated secondary antibody (Beyotime, China) for 1 h at room temperature. The fluorescent images were analyzed with Image J (NIH).

### Isolation and culture of primary mouse hippocampal neurons and microglia

Hippocampal neurons were isolated from embryonic day 17.5–18.5 mice. Cell suspensions were prepared and incubated in DMEM-F12 complete medium supplemented with 10% fetal bovine serum (FBS) and 1% penicillin–streptomycin for 24 h. The medium was subsequently replaced with serum-free DMEM-F12 supplemented with 2% B27, 1% penicillin–streptomycin, and 5 μmol/L cytarabine. The medium was refreshed 48 h later, followed by half-volume changes performed every 2 days thereafter.

For microglia culture, brains were harvested from postnatal day 1 (P1) mice to prepare cell suspensions. Cells were seeded in DMEM-F12 medium with 10% FBS and 1% penicillin–streptomycin, cultured for 24 h, and the medium was changed every 3 days. On days 10–12, microglia were purified by shaking the culture flask at 200 rpm for 4 h. The supernatant containing detached microglia was collected, centrifuged, and resuspended in fresh medium. Purified primary microglia (purity > 90%) were further cultured for 48 h.

### Lentivirus-mediated transfection

*Hmgb1* knockdown (KD) in primary neurons/microglia was achieved using CRISPR/Cas9. Mouse *Hmgb1*-specific sgRNA (5’-GGTGAGTGGTGATAAGCTGA-3’) and scramble sgRNA were cloned into the lentiCRISPR v2 vector (GeneChem, China). Lentiviruses were packaged in 293 T cells using psPAX2 and pMD2.G vectors. Cells were infected with lentivirus (MOI = 10) plus 8 μg/ml Polybrene, and positive clones were selected with 2 μg/ml puromycin for 7 days. KD efficiency (> 90%) was verified via Western blot (Fig. S4).

*Nrf2* overexpression (OE) was performed using the pLVX-Puro-*Nrf2* vector (GeneChem, China). Lentiviruses were packaged as described above, and cells were infected (MOI = 5) for 48 h. NRF2 protein overexpression (> twofold) was confirmed by Western blot, with an empty vector as the control (Fig. S4).

### Preparation of conditioned medium and simulated MetS microenvironment

To simulate the lipotoxic and glucotoxic microenvironment characteristic of MetS in vitro, neurons or microglia were first cultured in normal medium (NM) or high-fat high-glucose culture medium (HFHGM, containing 120 μM palmitic acid and 35 mM glucose) for 24 h. This combined exposure paradigm is a well-established in vitro model that reliably recapitulates the core metabolic stress features (i.e., glucolipotoxicity) driving neuronal injury and neuroinflammation in MetS, as demonstrated in multiple previous studies [51, 52]. The specific concentrations of palmitic acid and glucose were determined based on these previous protocols and further validated by our preliminary dose–response cell viability assays (Fig. S3). These conditions were selected to reliably induce metabolic stress-associated pathological changes while preserving sufficient cell viability for subsequent mechanistic and co-culture assays. Supernatants were collected, centrifuged and filtered to remove cellular debris, yielding conditioned medium (CM) for subsequent treatments. Recipient microglia or neurons were incubated with the prepared CM for 24 h.

### Cell treatments

For HMGB1 Ab or rHMGB1 treatment, cells were incubated with 5 μg/ml HMGB1 Ab (Puer Biotech, China) or 100 ng/ml rHMGB1 (FineTest, China), respectively. For Lip-1 treatment, neurons were treated with 200 nM Lip-1 (APExBio, USA). Regarding Minocycline processing, microglia were treated with 20 μM minocycline. HCQ (Selleck, USA) was administered at 3 μM for microglia and 1 μM for neurons. To block specific HMGB1 receptors, primary neurons were pre-treated with 10 μM TLR4 inhibitor TAK-242 (MedChemExpress, USA) or 3 μM RAGE inhibitor FPS-ZM1 (MedChemExpress, USA) for 1 h prior to HFHGM exposure.

### Measurement of Fe^2^⁺ and MDA

Hippocampal tissues and neurons were lysed. The Fe^2^⁺ and MDA levels were measured using commercial kits (Fe^2^⁺: Elabscience; MDA: Beyotime) following the manufacturers’ protocols.

### Enzyme-linked immunosorbent assay (ELISA)

ELISA kits specific for HMGB1 (Thermo Fisher), TNF-α, and IL-6 (Shanghai Enzyme-linked Biotechnology, China) were employed to quantify the levels of these analytes in hippocampal homogenates or culture supernatants, following the manufacturers’ protocols.

### Western blot analysis

Hippocampal tissues or cells were lysed. Nuclear/cytosolic fractions were isolated using a Nuclear and Cytoplasmic Extraction kit (Beyotime, China). Protein concentration was quantified via BCA assay.The proteins were separated by SDS polyacrylamide gel electrophoresis and transferred to the nitrocellulose membrane. The membranes were then blocked with 5% nonfat milk and immunoblotted. The protein bands were detected with an ECL kit (Beyotime Biotechnology, China). The following antibodies were used: anti-ACSL4 (Abcam, UK), anti-HMGB1 (Abcam, UK), anti-NRF2 (Abcam, UK), anti-ubiquitin (Abcam, UK), anti-LC3-II (Abcam, UK), anti-p62 (Abcam, UK), and anti-β-actin (Proteintech, USA). Protein bands were visualized using a chemiluminescence imaging system, and densitometric quantification was performed using ImageJ software (NIH, USA). For the cycloheximide (CHX) chase assay, cells were exposed to 50 μg/ml CHX, harvested at 0, 15, 30 and 60 min post-treatment, and analyzed via Western blot to evaluate NRF2 protein stability.

### Cell viability assay

Neuronal viability was determined using the Cell Counting Kit-8 (CCK-8; Beyotime Biotechnology, China) according to the manufacturer’s instructions.

### Real-time quantitative PCR (RT-PCR)

Total RNA was extracted using TRIzol reagent (Thermo Fisher). Primers (synthesized by GenScript, China; sequences in Table S1) were used for reverse transcription with a premix kit (Accurate Biology, China). Real-time quantification was performed on the ABI Q6 detection system (Applied Biosystems, USA). Target gene expressions, including *Aif1* (encoding IBA-1), *Tnf*, and *Il6*, were normalized to the reference gene.

### Co-Immunoprecipitation (Co-IP)

For Co-IP analysis, cells were first lysed in IP buffer (25 mM Tris–HCl pH 7.4, 150 mM NaCl, 1% NP-40) supplemented with protease and phosphatase inhibitors. Lysates were incubated with 2 μg anti-NRF2 antibody or IgG control at 4 °C for 4 h, then mixed with 20 μl Protein A/G Agarose (Beyotime, China) overnight. Beads were washed five times with IP buffer, boiled in 2 × loading buffer, and analyzed via Western blot with anti-ubiquitin antibody. To detect ubiquitinated NRF2, cells were exposed to 10 μM MG132 for 2 h to inhibit protein degradation.

### Statistical analysis

Statistical analysis was performed using GraphPad PRISM 9.4.0 (San Diego, California, USA). Normality was tested via the Shapiro–Wilk test, and variance homogeneity via Levene’s test. Group differences were compared using one-way analysis of variance (ANOVA) followed by Bonferroni post-hoc tests. All in vitro experiments were independently repeated at least three times, and in vivo data were collected from appropriate sample sizes as explicitly stated in the respective figure legends. Data were presented as mean ± standard deviation (SD), with significance set at *p* < 0.05 (*p < 0.05, **p < 0.01, ***p < 0.001).

## Supplementary Information

Supplementary Material 1.

## Data Availability

The data that support the findings of this study are available upon request.
