# Supplementary material for: HMGB1 aggravates neuronal ferroptosis and microglial inflammation in metabolic syndrome-associated cognitive impairment via the NRF2-autophagic flux axis
Source: Mol Biomed. 2026 Aug 18;7:141. doi: 10.1186/s43556-026-00548-8 (PMC13481606; doi:10.1186/s43556-026-00548-8)
Supplement: Supplementary file 1 [file 43556_2026_548_MOESM1_ESM.pdf]

Supplementary Material for

HMGB1 aggravates neuronal ferroptosis and microglial inflammation in metabolic syndrome-associated cognitive impairment via the NRF2-autophagic flux axis

Xin Zhang [1,2†], Yue Zhang [3,4†], Meng-Meng Tang [1], Jia-Mei Zhang [1], Ya-Qin Xiao[3], Jian-Jun Yang[1,5*], Cun-Ming Liu[1,*] and Min Yu [1,*]

[1] Department of Anesthesiology and Perioperative Medicine, The First Affiliated Hospital of Nanjing Medical University, Nanjing 210029, China

[2] Department of Anesthesiology, The Fourth Affiliated Hospital of Nanjing Medical University, Nanjing 210031, China

[3] Department of Anesthesiology, Jinling Hospital, Medical School of Nanjing University, Nanjing 210002, China

[4] Department of Anesthesiology, Jiangsu Province Academy of Traditional Chinese Medicine, Nanjing 210008, China

[5] Department of Anesthesiology, Pain and Perioperative Medicine, The First Affiliated Hospital of Zhengzhou University, Zhengzhou, 450000, China

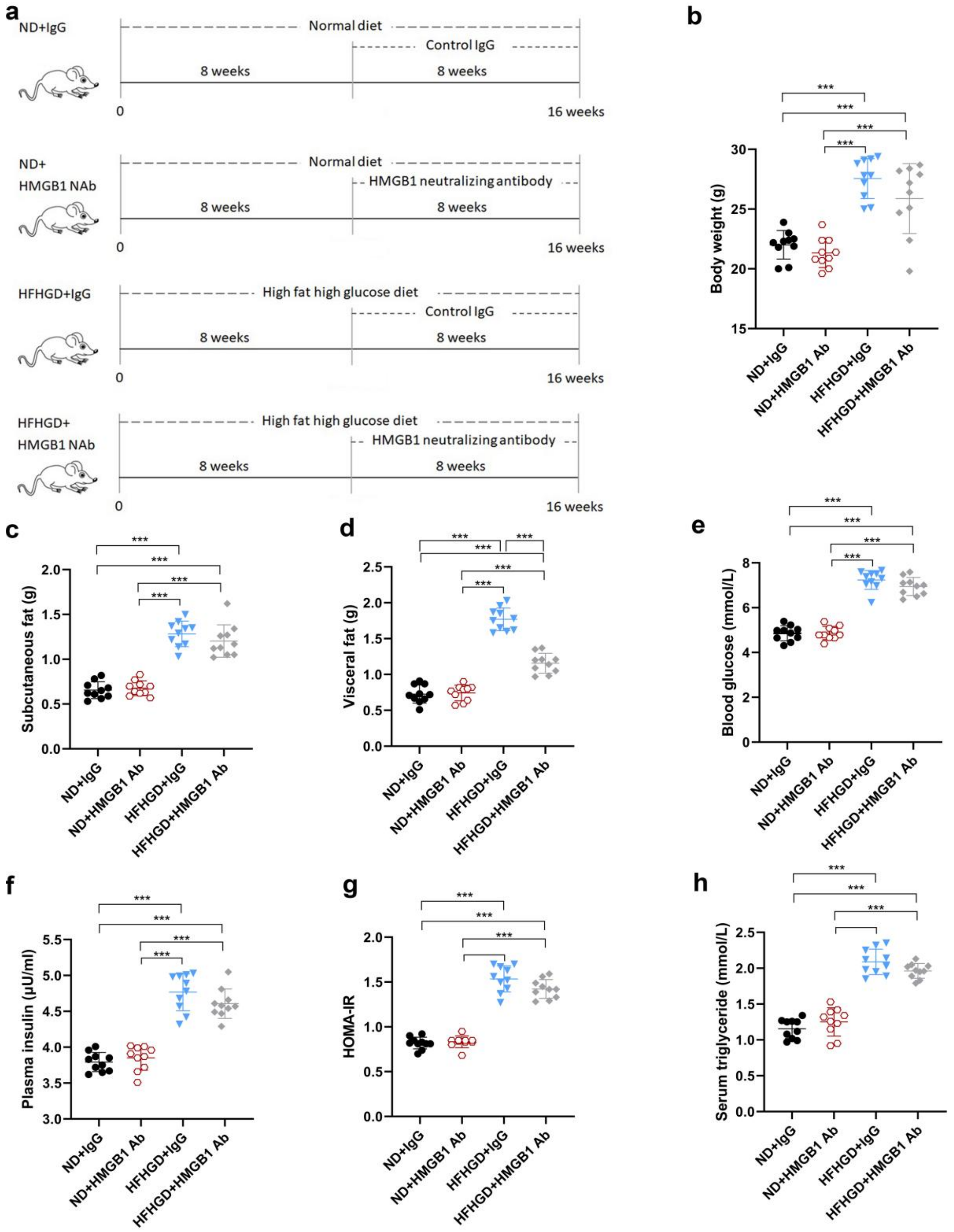

Fig. S1 Experimental design and validation of the metabolic syndrome (MetS) mouse model. **a** Schematic diagram of the *in vivo* experimental design and HMGB1 neutralizing antibody intervention timeline (n = 10 mice per group). **b-h** Metabolic parameters were assessed in C57BL/6J mice fed a normal diet (ND) or a high-fat high-glucose diet (HFHGD) for 16 weeks, including body weight (**b**), subcutaneous fat weight (**c**), visceral fat weight (**d**), fasting blood glucose (**e**), plasma insulin (**f**), HOMA-IR index (**g**), and serum triglyceride levels (**h**). Data for metabolic parameters are presented as mean ± SD (n = 10 mice per group). Statistical significance was evaluated using an unpaired Student's t-test. **$P < 0.001$, $P < 0.01$, $P < 0.05$.

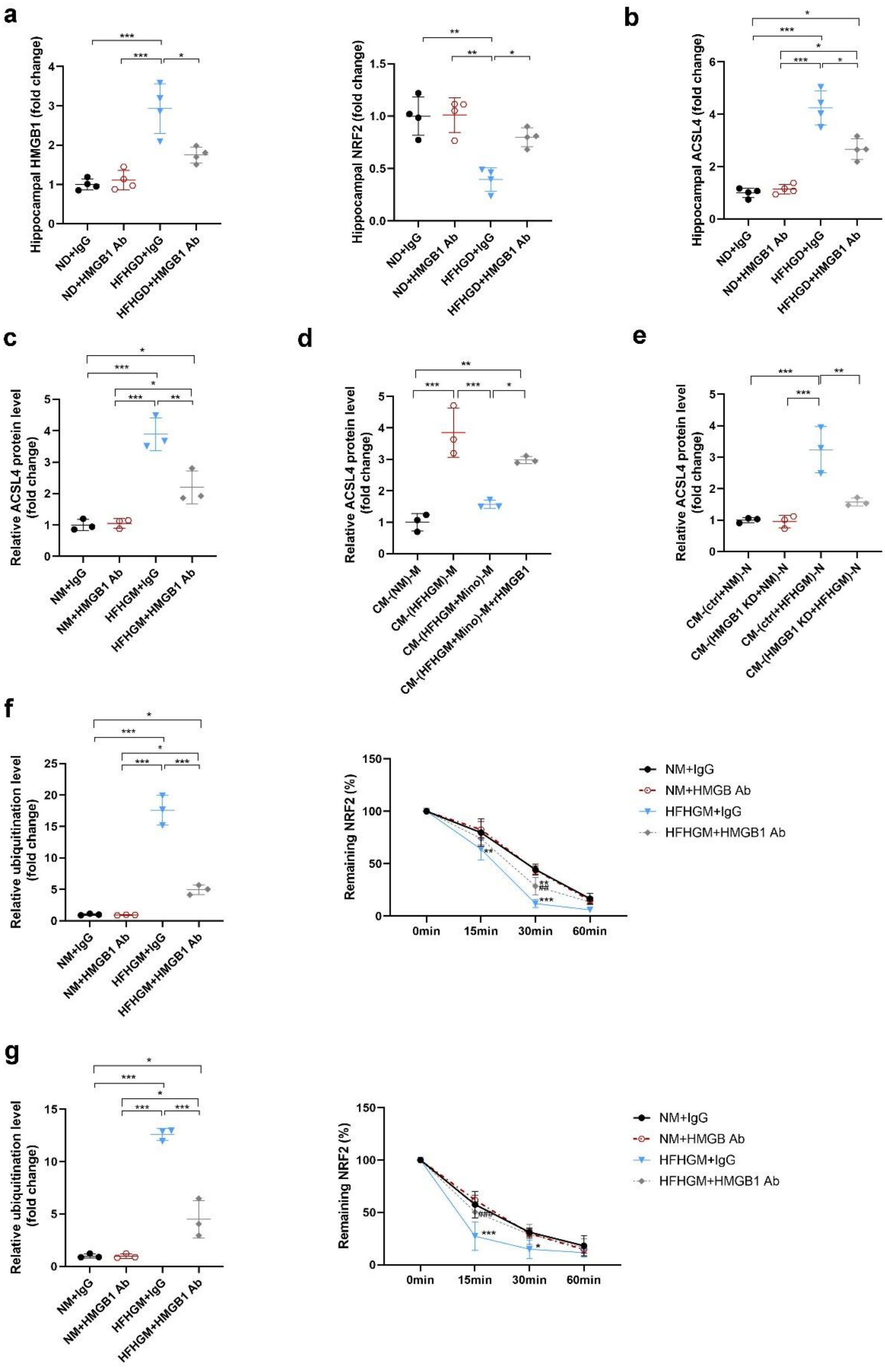
a
Hippocampal HMGB1 (fold change)
Hippocampal NRF2 (fold change)
ND+IgG
ND+HMGB1 Ab
HFHGD+IgG
HFHGD+HMGB1 Ab
b
Hippocampal ACSL4 (fold change)
c
Relative ACSL4 protein level (fold change)
NM+IgG
NM+HMGB1 Ab
HFHGM+IgG
HFHGM+HMGB1 Ab
d
CM-(NM)-M
CM-(HFHGM)-M
CM-(HFHGM+Mino)-M
CM-(HFHGM+Mino)-M+rHMGB1
e
CM-(ctrl+NM)-N
CM-(HMGB1 KD+NM)-N
CM-(ctrl+HFHGM)-N
CM-(HMGB1 KD+HFHGM)-N
f
Relative ubiquitination level (fold change)
Remaining NRF2 (%)
0min
15min
30min
60min
NM+IgG
NM+HMGB Ab
HFHGM+IgG
HFHGM+HMGB1 Ab
g

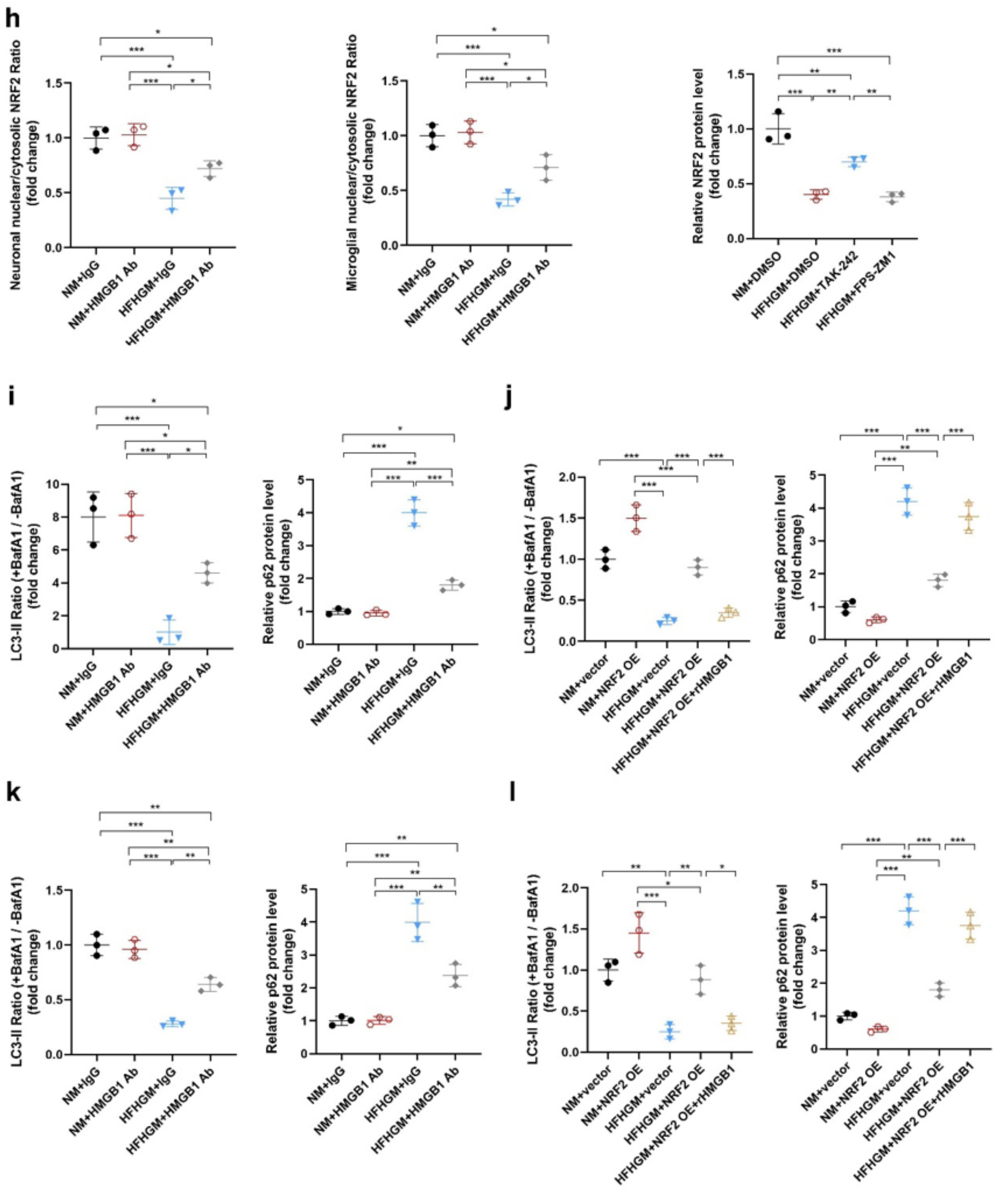

Fig. S2 Densitometric quantification of all Western blots and Co-IP assays. **a** Quantitative analysis of hippocampal HMGB1 and NRF2 protein levels in the *in vivo* MetS mouse model (**corresponding to Fig. 1a, b**). Data are presented as mean ± SD with individual data points (n=4 mice per group). **b** Quantitative analysis of ACSL4 protein expression in the hippocampus (**corresponding to Fig. 1g**). Data are presented as mean ± SD (n=4 mice per group). **c** Quantitative analysis of ACSL4 protein expression in primary neurons co-cultured with microglia under HFHGM conditions (**corresponding to Fig. 3e**). Data are presented as mean ± SD (n=3 independent experiments). **d, e** Quantitative analysis of ACSL4 protein expression in primary neurons treated with microglia-derived conditioned medium. Panel **d** corresponds to the minocycline and rHMGB1 rescue assay (**Fig. 5e**), and panel **e** corresponds

to the microglial *Hmgb1*-KD assay (**Fig. 5i**). Data are presented as mean ± SD (n=3 independent experiments). **f, g** Quantitative analysis of NRF2 ubiquitination and protein stability in primary neurons (**f**, **corresponding to Fig. 6a, b**) and microglia (**g**, **corresponding to Fig. 6d, e**). Bar graphs represent the integrated density of the ubiquitin smear normalized to total immunoprecipitated NRF2. Degradation kinetics (CHX chase assay) are presented as line graphs plotting the percentage of remaining NRF2 protein relative to Time 0 over the indicated time course. Data are presented as mean ± SD (n=3). **h** Quantitative analysis of NRF2 nuclear translocation (Nuclear/Cytosolic ratio) and TLR4/RAGE receptor blockade assays in primary neurons (**corresponding to Fig. 6c, f, g**). Data are presented as mean ± SD (n=3). **i, j** Quantitative analysis of autophagic flux assessing LC3-II turnover and p62 accumulation in primary neurons treated with or without HMGB1 Ab (**i**, **corresponding to Fig. 7a**) or subjected to *Nrf2* OE with or without rHMGB1 (**j**, **corresponding to Fig. 7b**). **k, l** Quantitative analysis of autophagic flux (LC3-II and p62) in primary microglia treated with or without HMGB1 Ab (**k**, **corresponding to Fig. 7c**) or subjected to *Nrf2* OE with or without rHMGB1 (**l**, **corresponding to Fig. 7d**). Autophagic flux (LC3-II turnover) is presented as the ratio of LC3-II levels in the presence of BafA1 to those in the absence of BafA1. Basal p62 accumulation was quantified from the -BafA1 lanes. All densitometric analyses were performed using ImageJ software, with target protein signals normalized to their respective internal controls (β-actin for total/cytosolic proteins, Histone H3 or Lamin B1 for nuclear fractions). Statistical significance was evaluated using one-way ANOVA followed by Bonferroni post-hoc tests (* $P < 0.05$, ** $P < 0.01$, *** $P < 0.001$). For the CHX chase assay degradation kinetics (line graphs), a two-way ANOVA followed by Bonferroni post-hoc tests was employed to analyze the interaction between time and treatment groups (* $P < 0.05$, ** $P < 0.01$, *** $P < 0.001$ compared to control groups; # $P < 0.05$, ## $P < 0.01$, ### $P < 0.001$ compared to the corresponding HFHGM groups).

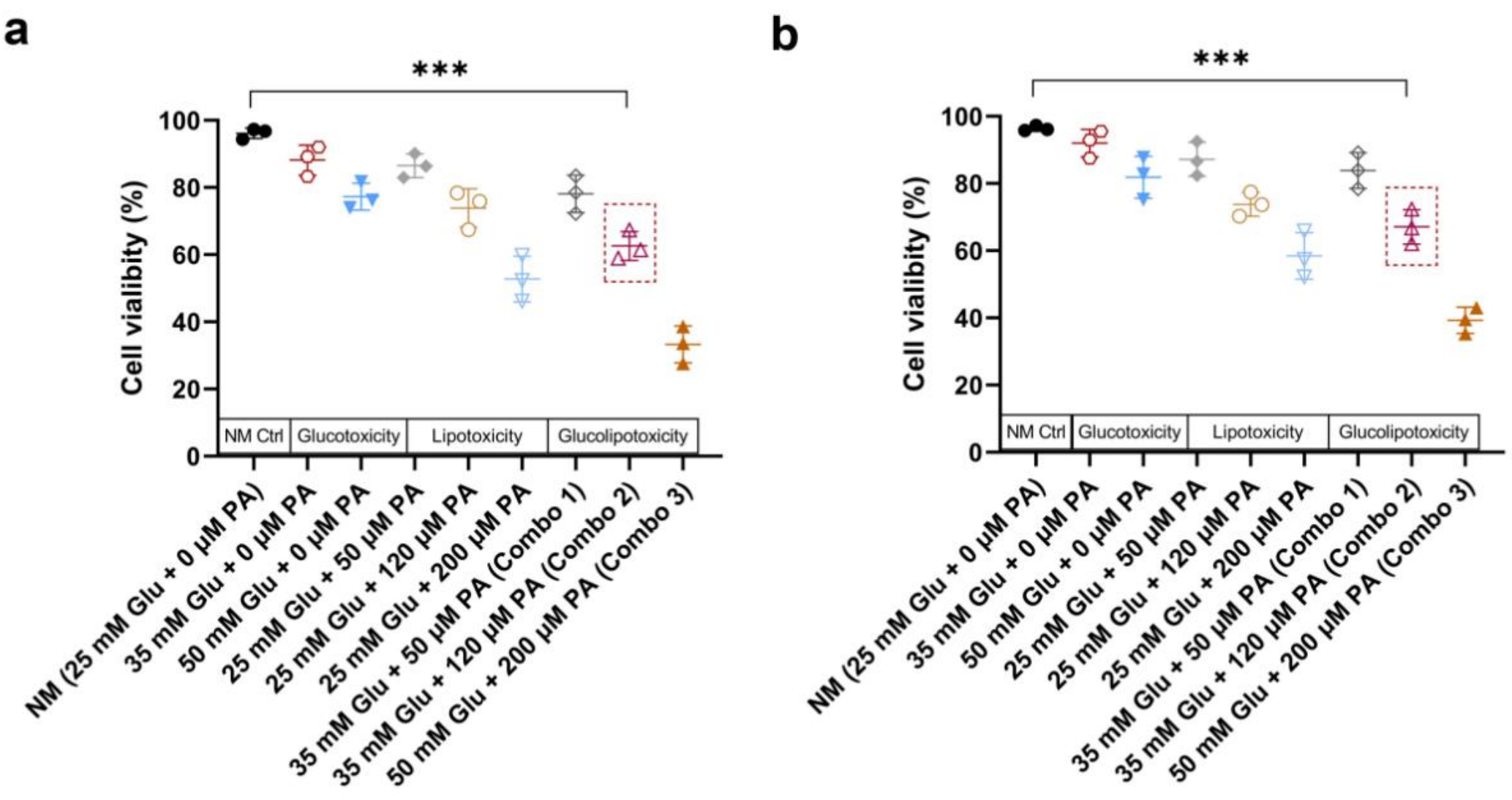

Fig. S3 Dose-response effects of palmitic acid and glucose on the viability of primary neurons and microglia. **a** Cell viability of primary mouse neurons exposed to varying concentrations of palmitic acid (PA) combined with different concentrations of glucose for 24 h, assessed by CCK-8 assay. **b** Cell viability of primary mouse microglia exposed to the same gradient combinations for 24 h. The basal normal medium (NM) contains 25 mM glucose. The combined condition of 120 µM PA and a total of 35 mM glucose (indicated by the red box) was selected as the HFHGM for subsequent experiments, as it robustly induced glucolipotoxicity (manifested as significant reduction in cell viability) without causing immediate massive cell death. Statistical significance was evaluated using one-way ANOVA followed by Bonferroni post-hoc tests. Data are presented as mean ± SD of three independent experiments (n = 3). * $P < 0.05$, ** $P < 0.01$, *** $P < 0.001$.

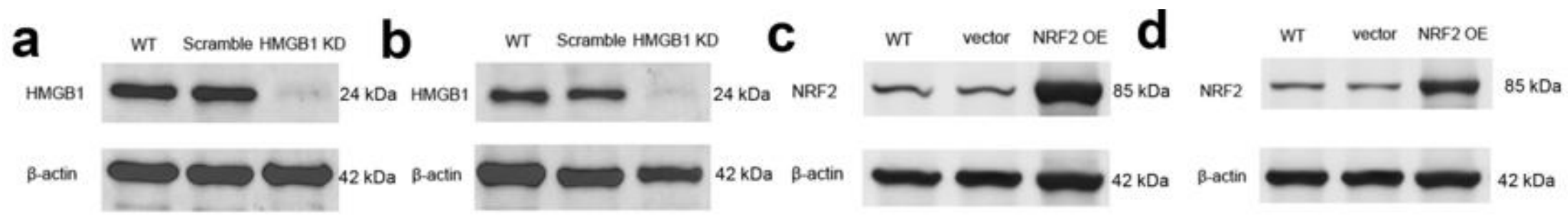

Fig. S4 Validation of *Hmgb1* knockdown (KD) and *Nrf2* overexpression (OE) efficiency. **a** Representative Western blot image of HMGB1 protein expression in neurons with or without *Hmgb1* knockdown. **b** Representative Western blot image of HMGB1 protein expression in microglia with or without *Hmgb1* knockdown. **c** Representative Western blot image of NRF2 protein expression in neurons with or without *Nrf2* overexpression. **d** Representative Western blot image of NRF2 protein expression in microglia with or without *Nrf2* overexpression. KO efficiency was >70%, and OE efficiency was >3-fold, confirming successful genetic manipulation.

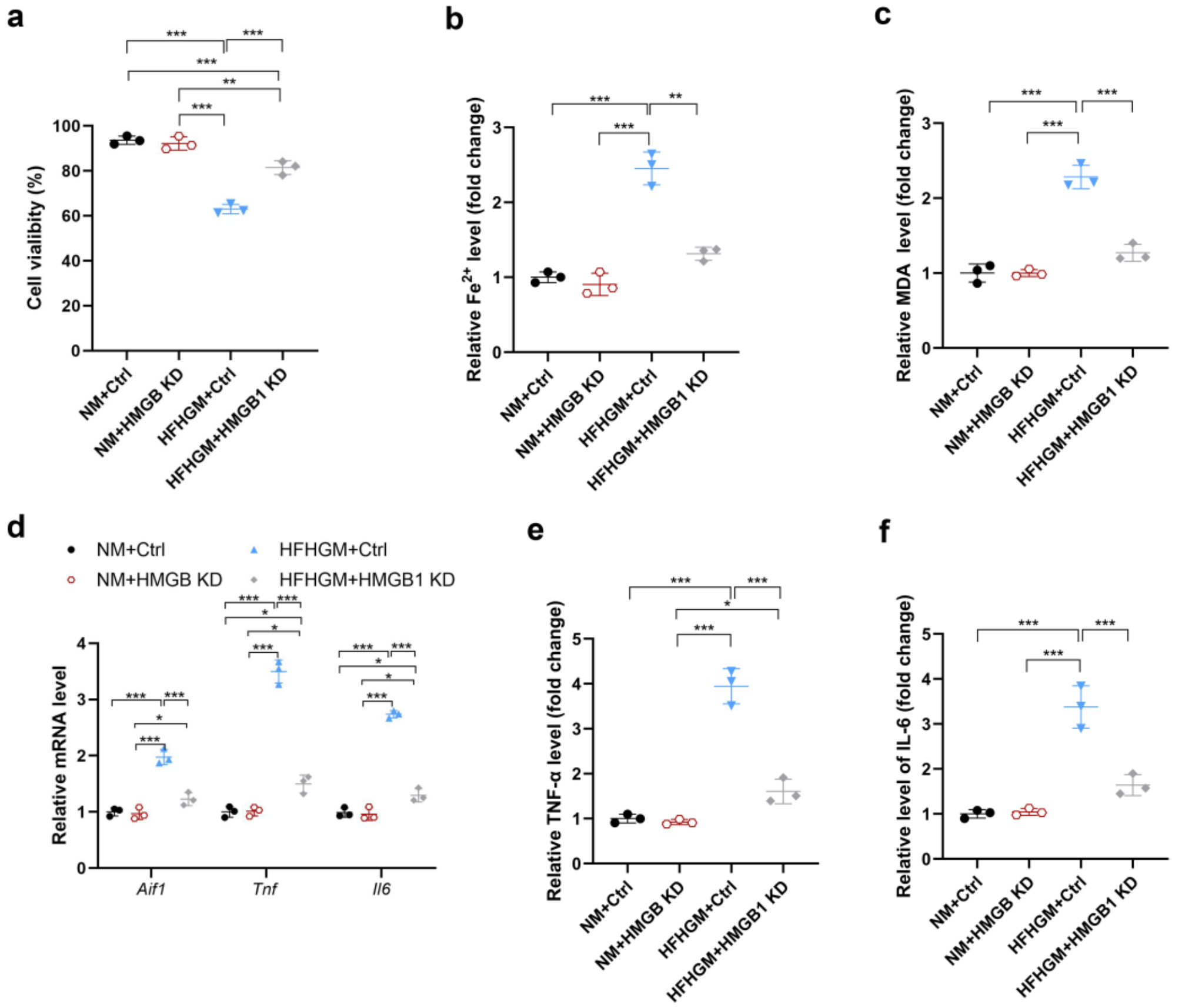

Fig. S5 HMGB1 knockdown reduces neuronal ferroptosis and microglial inflammation under HFHGM stress. **a, b, c** Viability (**a**), Fe^{2+} (**b**) and MDA (**c**) levels in neurons with or without HMGB1 knockdown. **d** Relative mRNA levels of *Aif1* (IBA-1), *Tnf* and *Il6* in microglia with or without HMGB1 knockdown. **e, f** Secreted TNF-α (**e**) and IL-6 (**f**) protein levels in culture medium of mouse microglia with or without HMGB1 knockdown. Data are presented as mean ± SD of three independent experiments (n=3). Statistical significance was evaluated using one-way ANOVA followed by Bonferroni post-hoc tests. * $P < 0.05$, ** $P < 0.01$, *** $P < 0.001$.

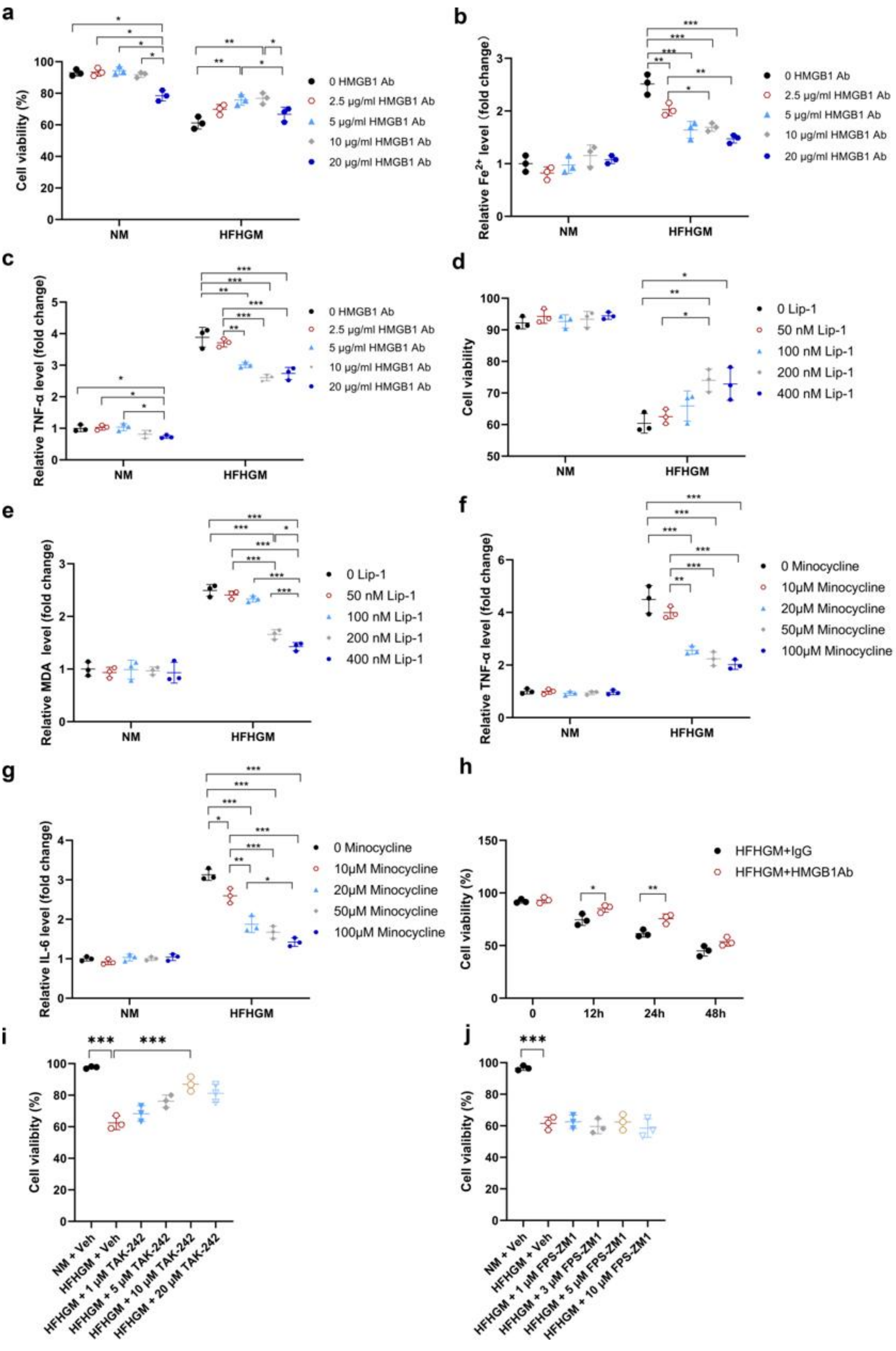

Fig. S6 Optimization of concentration and time-course for HMGB1 neutralizing antibody, ferroptosis/microglial inhibitors, and receptor antagonists. **a, b** Viability (**a**) and Fe^{2+} (**b**) level of neurons treated with 0, 2.5, 5, 10, 20 μg/mL HMGB1 Ab in NM or HFHGM for 24h. **c** Secreted TNF-α protein levels in medium of microglia treated with 0, 2.5, 5, 10, 20 μg/mL HMGB1 Ab in NM or

HFHGM for 24h. **d,e** Viability (**d**) and MDA (**e**) levels of neurons treated with 0, 50, 100, 200, 400 nM Lip-1 in NM or HFHGM for 24h. **f, g** Secreted TNF-α (**f**) and IL-6 (**g**) protein levels in medium of microglia treated with 0, 10, 20, 50, 100 μM Minocycline in NM or HFHGM. **h** Viability in neurons treated with HMGB1 Ab (5 μg/mL) for 0, 12, 24, 48 h in HFHGM. **i** Viability of primary neurons pre-treated with varying concentrations of the TLR4 inhibitor TAK-242 (0, 1, 5, 10, and 20 μM) followed by HFHGM exposure for 24 h. **j** Viability of primary neurons pre-treated with varying concentrations of the RAGE inhibitor FPS-ZM1 (0, 1, 3, 5, and 10 μM) followed by HFHGM exposure for 24 h. The optimal concentrations for TAK-242 (10 μM, providing maximal rescue without cytotoxicity) and FPS-ZM1 (3 μM, reflecting a standard working concentration despite lacking rescue efficacy) were established based on these dose-response curves and prior literature. Data are presented as mean ± SD of three independent experiments (n=3). Statistical significance for **a-g, i,** and **j** was evaluated using one-way ANOVA followed by Bonferroni post-hoc tests; significance for **h** was evaluated using multiple unpaired t-tests with Holm-Šídák correction. * $P < 0.05$, ** $P < 0.01$, *** $P < 0.001$.

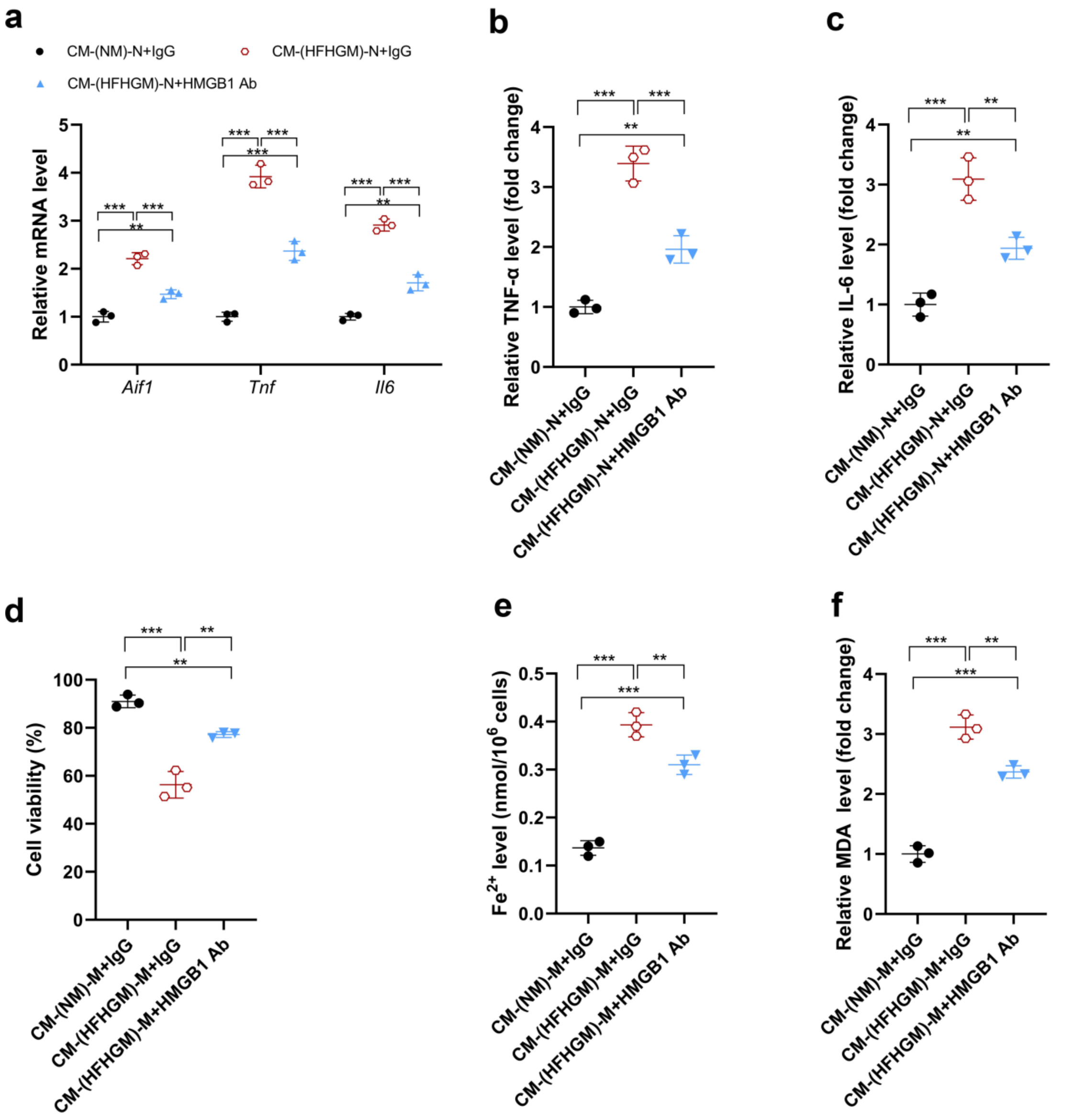

Fig. S7 HMGB1 neutralizing antibody attenuates the reciprocal aggravation in conditioned medium (CM) models. **a** Relative mRNA levels of *Aif1* (*IBA-1)*, *Tnf* and *Il6* in microglia treated with conditioned medium (CM) from NM- or HFHGM-processed neurons, or HFHGM-processed neuron-derived CM supplemented with HMGB1 Ab. **b, c** Secreted TNF-α (**b**) and IL-6 (**c**) protein levels in culture medium of microglia treated with CM from NM- or HFHGM-processed neurons, or HFHGM-processed neuron-derived CM supplemented with HMGB1 Ab. **d** Viability of neurons treated with CM from NM- or HFHGM-processed microglia, or HFHGM-processed microglia-derived CM supplemented with HMGB1 Ab. **e, f** Fe^{2+} (**e**) and MDA (**f**) levels in neurons treated with CM from NM- or HFHGM-processed microglia, or HFHGM-processed microglia-derived CM supplemented with HMGB1 Ab. Data are presented as mean ± SD of three independent experiments (n=3). Statistical significance was evaluated using one-way ANOVA followed by Bonferroni post-hoc tests. * $P < 0.05$, ** $P < 0.01$, *** $P < 0.001$.

Table S1 Primer sequences for quantitative real-time PCR (qRT-PCR)

All primers were designed and synthesized by GenScript (China). Sequences are listed as follows:

Target	Species	Forward Primer (5'→3')	Reverse Primer (5'→3')
Aif1 (IBA-1)	Mouse	AATGATGAGGATCTGCCGTCCAAAC	TCTCTTCAGCTCTAGGTGGGTCTTG
Tnf	Mouse	CCCTCCAGAAAAGACACCATG	GCCACAAGCAGGAATGAGAG
Il6	Mouse	TTCTTGGGACTGATGCTGGTG	CACAACTCTTTTCTCATTTCCACGA
Actb (β-ACTIN)	Mouse	CTGAGAGGGAAATCGTGCGT	CCACAGGATTCCATACCCAAGA
